# Nonredundant, isoform-specific roles of HDAC1 in glioma stem cells

**DOI:** 10.1172/jci.insight.149232

**Published:** 2021-09-08

**Authors:** Costanza Lo Cascio, James B. McNamara, Ernesto L. Melendez, Erika M. Lewis, Matthew E. Dufault, Nader Sanai, Christopher L. Plaisier, Shwetal Mehta

**Affiliations:** 1 Ivy Brain Tumor Center, Barrow Neurological Institute, Phoenix, Arizona, USA.; 2 Interdisciplinary Graduate Program in Neuroscience, School of Life Sciences, and; 3 School of Biological and Health Systems Engineering, Arizona State University, Tempe, Arizona, USA.

**Keywords:** Oncology, Stem cells, Brain cancer, Cancer, Epigenetics

## Abstract

Glioblastoma (GBM) is characterized by an aberrant yet druggable epigenetic landscape. One major family of epigenetic regulators, the histone deacetylases (HDACs), are considered promising therapeutic targets for GBM due to their repressive influences on transcription. Although HDACs share redundant functions and common substrates, the unique isoform-specific roles of different HDACs in GBM remain unclear. In neural stem cells, HDAC2 is the indispensable deacetylase to ensure normal brain development and survival in the absence of HDAC1. Surprisingly, we find that HDAC1 is the essential class I deacetylase in glioma stem cells, and its loss is not compensated for by HDAC2. Using cell-based and biochemical assays, transcriptomic analyses, and patient-derived xenograft models, we find that knockdown of HDAC1 alone has profound effects on the glioma stem cell phenotype in a p53-dependent manner. We demonstrate marked suppression in tumor growth upon targeting of HDAC1 and identify compensatory pathways that provide insights into combination therapies for GBM. Our study highlights the importance of HDAC1 in GBM and the need to develop isoform-specific drugs.

## Graphical abstract

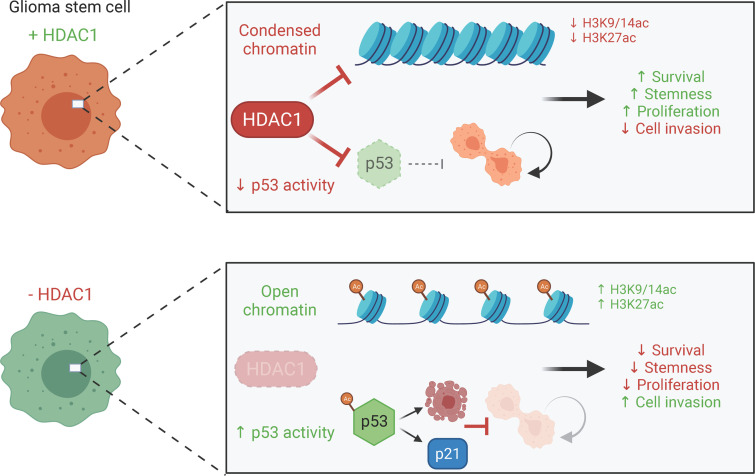

## Introduction

A well-regulated interplay between histone acetylation and deacetylation, mediated by histone acetyltransferases and histone deacetylases (HDACs), is essential for the dynamic fine-tuning of gene expression (1). HDACs mediate chromatin compaction, favoring transcriptional repression, and are frequently overexpressed in human cancers, including glioblastoma (GBM; refs. 2, 3). Hence, over the last decade, there has been considerable interest in HDAC inhibitors (HDACi) in the field of oncology. However, systemic inhibition of HDACs with pharmacological inhibitors fails to provide significant therapeutic benefit in clinical trials for primary and recurrent GBM (4–8). HDACi currently in clinical trials have poor brain penetration and a narrow therapeutic window due to their relatively low selectivity for individual HDAC isoforms (3, 7, 9–11). Notably, not all HDACs are equally expressed in GBM, and the specific functions of individual HDAC isoforms in these tumors are not well understood (12). Hence, there is a need to dissect the functional importance and requirement for individual HDACs in GBM, especially the therapy-resistant glioma stem cells (GSCs), to provide a rationale for the development of isoform-selective HDACi.

While it is known that HDAC1 and HDAC2 (class I HDACs) harbor highly specific and nonoverlapping roles in the developing brain, it is unclear whether these nonredundant functions are retained in glioma cells (2, 13). Our current knowledge on the role of HDACs in GBM is primarily based on pan-HDACi studies and isoform-specific knockdown or knockout experiments in serum-grown, long-term cultures of GBM cells and not in GSCs (14–20). Even the Cancer Dependency Map project, which profiled hundreds of cancer cell lines to identify genetic and pharmacological vulnerabilities, utilized traditionally grown glioma cell lines (20). Considering that GBMs frequently hijack normal developmental programs to their advantage and the fact that there is an isoform-specific requirement for HDAC2 over HDAC1 during normal brain development, we questioned whether GSCs maintain similar or divergent isoform-specific requirements for class I HDAC activity to sustain tumor growth (21–24).

Here, we demonstrate that HDAC1 is critical for the proliferative potential of GSCs in a p53-dependent manner. Knockdown of *HDAC1* alone results in significantly prolonged survival in a patient-derived xenograft (PDX) model and a mouse model of human glioma, and the resulting tumors exhibit a more invasive growth pattern. Our data reveal an essential nonredundant role of HDAC1 in GSCs in contrast to its dispensability in normal neural stem cells (21). This molecular selectivity for HDAC1 was evident upon its ablation in human GSCs (hGSCs), where its loss was not compensated by upregulation of its paralogue HDAC2 or other HDACs. Furthermore, we observe that ablation of HDAC1 function in GSCs suppressed expression of key glioma stemness markers like SRY-box transcription factor 2 (SOX2), Nestin, and oligodendrocyte transcription factor 2 (OLIG2). In addition, *HDAC1* silencing stabilized and acetylated p53, resulting in upregulation of key p53 target genes and induction of programmed cell death. This demonstrates that HDAC1, but not HDAC2, functions as a p53 deacetylase in hGSCs, as previously shown in several normal and tumorigenic cell lines (25–27). Our results uncover a previously underappreciated role for HDAC1 in regulating the GSC phenotype, and highlight opportunities for the development of isoform-specific HDACi for novel targeted and combinatorial therapies in GBM.

## Results

### HDAC1 expression is correlated with worse survival in GBM.

**Figure 1:**
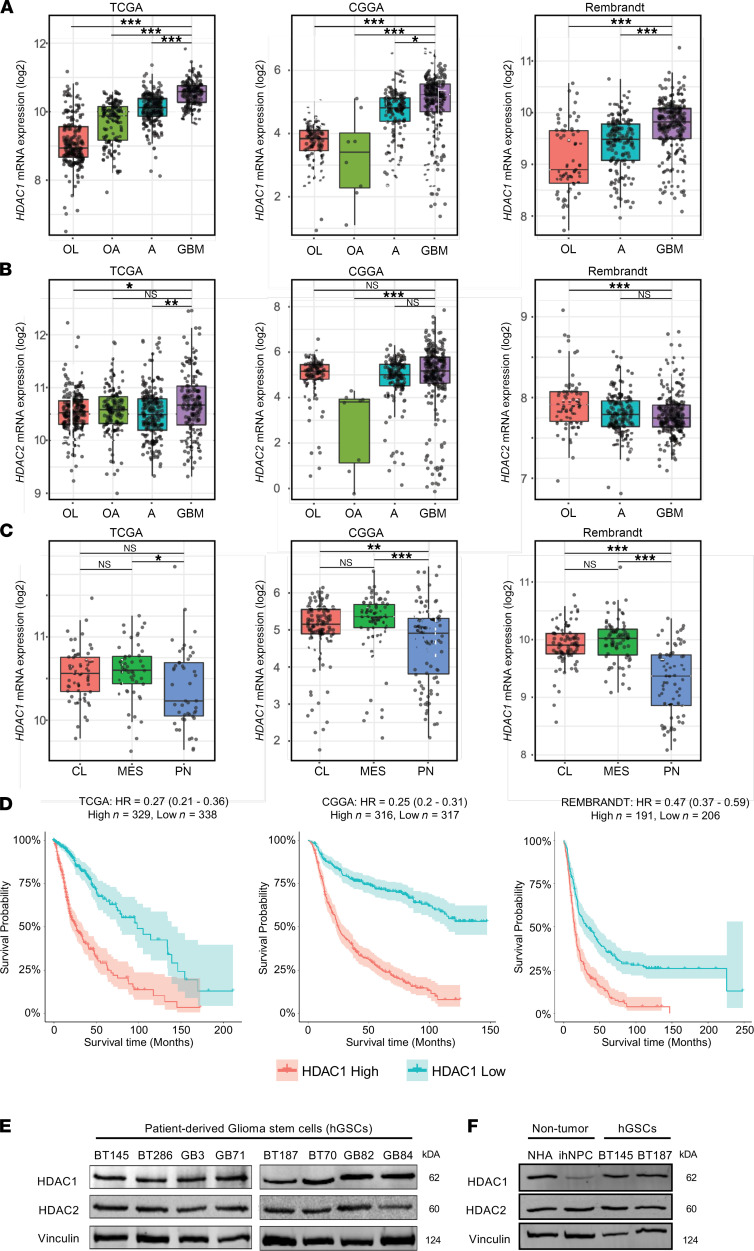
*HDAC1* expression levels in GBM. (**A**) *HDAC1* (**B**) and *HDAC2* expression levels across various grades of gliomas (OL, oligodendroglioma; OA, oligoastrocytoma; A, astrocytoma; GBM, glioblastoma) within the TCGA, CCGA, and REMBRANDT databases. *HDAC1*, but not *HDAC2*, expression significantly increases with malignancy; Tukey’s post hoc test. (**C**) *HDAC1* expression levels across the 3 GBM molecular subtypes (CL, classical; MES, mesenchymal; PN, proneural). (**D**) Kaplan-Meier analysis stratifying glioma patients with *HDAC1* high and low expression within the TCGA, CCGA, and Rembrandt databases; log-rank test. (**E**) Immunoblot showing basal levels of HDAC1 and HDAC2 in p53-WT hGSCs (BT145, BT286, GB3, and GB71) and p53-mutant hGSCs (BT187, BT70, GB82, and GB84) (*n* = 3). (**F**) Immunoblot showing basal levels of HDAC1 and HDAC2 in nontumorigenic normal human astrocytes (NHAs) and induced pluripotent stem cell–derived (iPSC-derived) human neural progenitor cells (ihNPCs) alongside 2 hGSC lines (*n* = 3). The box plots depict the minimum and maximum values (whiskers), the upper and lower quartiles, and the median. The length of the box represents the interquartile range. *P < 0.05; **P < 0.01; ***P < 0.001. See also Supplemental Figure 1.

We evaluated relative mRNA expression levels of *HDAC1* and *HDAC2* across different grades of glioma using the The Cancer Genome Atlas (TCGA), Chinese Glioma Genome Atlas (CGGA), and Repository of Molecular Brain Neoplasia Data (REMBRANDT) databases (12, 28, 29). *HDAC1* expression increased with WHO tumor grade, and its expression was significantly higher in grade IV (GBM) than in lower grade gliomas (*P* < 0.001; Figure 1A). In contrast, *HDAC2* was expressed at high levels across all glioma samples (Figure 1B). *HDAC1* expression does not differ significantly across the 3 molecular subgroups of GBM (ref. 30 and Figure 1C), and its expression was higher in GBM compared with normal brain tissue (Supplemental Figure 1A; supplemental material available online with this article; https://doi.org/10.1172/jci.insight.149232DS1). We also found that the expression of *HDAC1* was inversely correlated with patient survival in 3 independent data sets that include GBM and low-grade gliomas (Figure 1D and ref. 31). We compared HDAC1 and HDAC2 protein expression in gliomas using the Human Proteome Atlas and found that both were strongly expressed in most gliomas, although HDAC1 expression is higher in GBM than in low-grade gliomas (32). One caveat with these analyses is that they were conducted on bulk GBM tissue and thus do not provide information on the functions of these 2 HDAC paralogues within the GSC population. Hence, given these expression data and the role of HDAC1 as a global repressor of transcription (13), we sought to specifically understand its functional impact across multiple patient-derived GBM GSCs (hGSCs).

### HDAC1 and HDAC2 are abundantly expressed in hGSCs.

We compared the expression of HDAC1 and HDAC2 across 8 hGSC lines (Figure 1E), normal human astrocytes (NHAs), and human neural progenitor cells derived from induced pluripotent stem cells from a healthy individual (ihNPCs; Figure 1F). HDAC1 and HDAC2 were equally and abundantly expressed in NHAs and hGSC lines (Figure 1, E and F), while HDAC1 levels were lowest in normal ihNPCs (Figure 1F). The latter observation is in line with previous reports that demonstrated that HDAC1 and HDAC2 display different lineage-specific or developmental stage expression patterns — with HDAC2 being more highly expressed than HDAC1 in neural precursors and mature neurons (21, 33). Another class I HDAC that has been previously implicated in GBM biology and whose expression correlates with increased glioma aggressiveness is HDAC3 (34, 35). However, we found that HDAC3 protein levels were very low compared with HDAC1 across multiple primary hGSC cell lines (see Supplemental Figure 1B). Hence, our goal was to investigate the significance of high HDAC1 expression in GBM tumors and assess whether hGSCs harbor differential dependencies or degrees of functional redundancy with respect to the activity of the 2 class I HDAC paralogues.

### HDAC1 knockdown suppresses viability of hGSCs in a p53-dependent manner.

**Figure 2:**
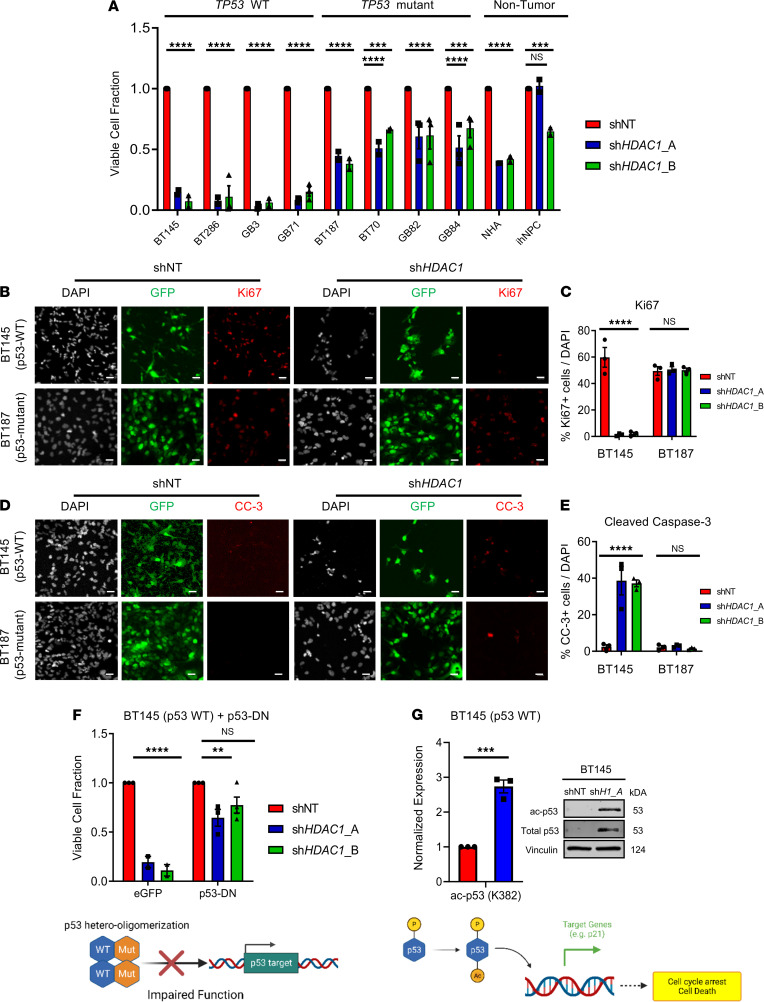
Knockdown of *HDAC1* reduces viability of hGSCs in a p53-dependent manner. (**A**) Quantification of the percentage of viable p53-WT and p53-mutant hGSCs and 2 nontumorigenic cell lines (NHAs, ihNPCs) transduced with sh*HDAC1*_A or sh*HDAC1*_B, compared with control cells transduced with nontarget shRNA (shNT) (*n* = 3). (**B** and **C**) Immunofluorescence staining (**B**) and quantification (**C**) of Ki67-positive hGSCs after acute *HDAC1* silencing (*n* = 3). (**D** and **E**) Immunofluorescence staining (**D**) and quantification (**E**) of cleaved caspase-3–positive cells after acute *HDAC1* silencing (*n* = 3). (**F**) Quantification of the percentage of viable p53-WT hGSCs overexpressing p53-DN or EGFP after *HDAC1* knockdown (*n* = 3). Schematic below illustrates how overexpression of a p53 mutant (p53-DN) affects p53 function. (**G**) Quantification of immunoblots for total and acetylated p53 (K382) after *HDAC1* silencing (sh*H1*_A, sh*HDAC1*_A) in p53-WT BT145 (*n* = 3). Schematic below illustrates how HDAC1 opposes p53 activation through direct deacetylation. For each cell line, the data are compiled from at least 3 independent experiments for each shRNA. Error bars indicate SEM. **P < 0.01, ***P < 0.001, ****P < 0.0001. Original magnification, 20×; scale bars, 2 μm. *P* values were determined using the 2-way ANOVA with Tukey’s multiple comparisons test or unpaired 2-tailed *t* test. See also Supplemental Figure 2, Supplemental Figure 3, and Supplemental Tables 1 and 2.

We silenced *HDAC1* expression in 4 distinct p53 wild-type (p53-WT) hGSC lines (BT145, GB3, BT286, and GB71), p53-mutant hGSC lines (BT187, BT70, GB82, GB84), NHAs, and ihNPCs using 2 independent shRNAs targeting *HDAC1* protein-coding regions (sh*HDAC1*_A and sh*HDAC1*_B). Acute knockdown of *HDAC1* resulted in a dramatic reduction in the viability of all 4 hGSC cultures (Figure 2A, Supplemental Figure 2A, and Supplemental Figure 3A; approximately 85%–90% loss in viability compared with controls; *P* < 0.0001). *HDAC1* knockdown had a significant but much more attenuated impact on the survival of p53-mutant hGSCs (average 50%–55% reduction; Figure 2A, Supplemental Figure 2B, Supplemental Figure 3B, and Supplemental Table 1; *P* < 0.001–0.0001). Two isocitrate dehydrogenase–mutant hGSCs harboring p53 mutations were practically unaffected by loss of HDAC1 (HK211, HK252; Supplemental Figure 2, E and F, and Supplemental Figure 3C). The viability of nontumorigenic NHAs and ihNPCs was also significantly affected by absence of HDAC1 protein, albeit not to the same extent as p53-WT hGSCs (Figure 2A, Supplemental Figure 2C, and Supplemental Figure 3D).

We assessed the proportion of actively cycling or dying cells after acute *HDAC1* knockdown through immunocytochemistry in both p53-WT and p53-mutant hGSCs (Figure 2, B–E). We found that in p53-WT cells (BT145), *HDAC1* knockdown led to a significant reduction in the percentage of Ki67-positive cells (>95% decrease; *P* < 0.001) and significant increase in the percentage of cleaved caspase-3–positive cells (average 37% increase; *P* < 0.01; Figure 2, B–E). Conversely, *HDAC1* knockdown did not affect proliferation and survival of p53-mutant cells (BT187) compared to nontarget controls (Figure 2, B–E). Hence, reduced proliferation and increased cell death contribute to the decrease in cell viability observed in p53-WT hGSCs. To confirm whether the effects of *HDAC1* knockdown on hGSC proliferation are indeed dependent on p53 status, we transduced a p53-WT hGSC line (BT145) with a dominant-negative form of *TP53* (p53-DN; ref. 36). As shown in Figure 2F, overexpression of p53-DN in BT145 rescued the defect in proliferation observed after *HDAC1* knockdown. Next, to assess the impact of HDAC1 on p53 activation, we examined acetylation status of p53 after HDAC1 knockdown. HDAC1 knockdown resulted in significantly increased acetylation of p53 at lysines 382 and 373 and its stabilization (Figure 2G and Supplemental Figure 3, E and F). These posttranslational modifications have been shown to be important for p53 stabilization, subsequent activation, and transcriptional activity (26, 37, 38). *HDAC1* knockdown did not result in increased transcription of *TP53* or decreased transcription of its negative regulator *MDM2* (Supplemental Figure 3G). These results indicate that HDAC1 regulates p53 acetylation and suppresses its transcriptional functions in p53-WT hGSCs and strongly suggest that p53 status dictates the response of hGSCs to acute loss of HDAC1 protein.

### HDAC1 function is nonredundant in hGSCs and is not compensated for by other HDACs.

**Figure 3:**
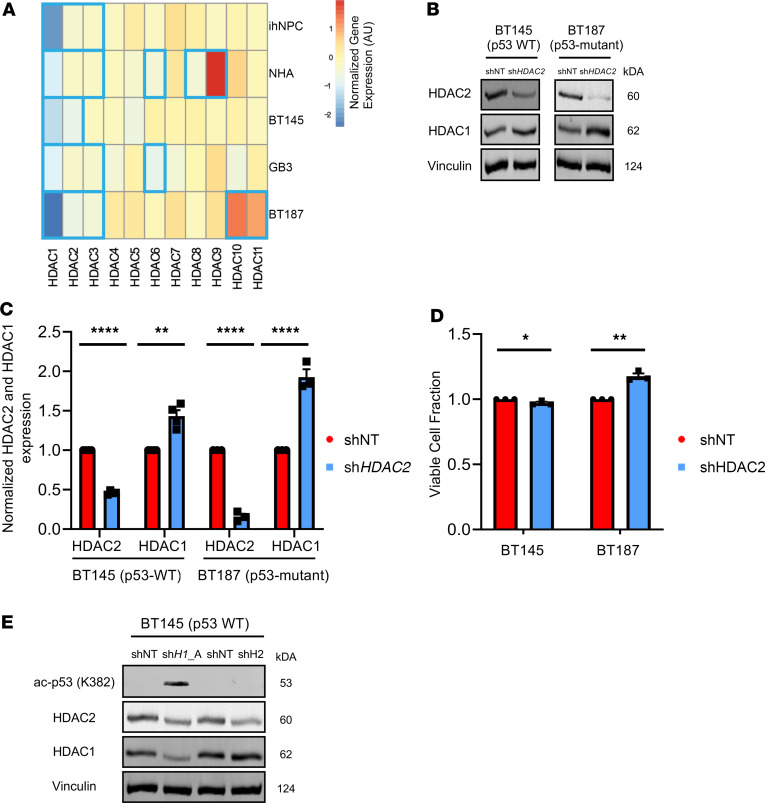
HDAC1 function is nonredundant in hGSCs and is not compensated for by its paralogue HDAC2. (**A**) Log_2_ fold change of differential expression for the 11 HDACs (HDAC1–11) after short hairpin *HDAC* (sh*HDAC1*) knockdown in 2 nontumorigenic (ihNPC and NHA) and 3 hGSC (BT145, GB3, BT187) cell lines. Blue bolded boxes indicate significant differential expression (adjusted *P* ≤ 0.05). (**B**) Representative immunoblot showing protein levels of HDAC1 and HDAC2 after acute *HDAC2* knockdown (sh*HDAC2*) in p53-WT (BT145) and p53-mutant (BT187) hGSCs. (**C**) Quantification of expression of HDAC2 and HDAC1 protein (normalized to Vinculin) after *HDAC2* knockdown in BT145 (*n* = 4) and BT187 (*n* = 3). (**D**) Quantification of the percentage of viable hGSCs (BT145 and BT187) 7 days after *HDAC2* knockdown, relative to shNT controls (*n* = 3). (**E**) Immunoblot comparing levels of acetylated p53 (K382) and HDAC1 and HDAC2 protein after *HDAC1* and *HDAC2* silencing in p53-WT hGSCs (BT145). For each cell line, the data are compiled from at least 3 independent experiments for each shRNA. Error bars indicate SEM. *P < 0.05, **P < 0.01, ****P < 0.0001. *P* values were calculated using unpaired 2-tailed *t* test. See also Supplemental Figure 4.

We sought to determine the global transcriptional consequences of HDAC1 loss and obtain insights into the mechanisms underlying the distinct phenotypes exhibited by hGSCs and normal neural cells. We performed RNA-sequencing (RNA-Seq) analysis of p53-WT (BT145, GB3) and p53-mutant (BT187) hGSCs as well as NHAs and ihNPC cultures after *HDAC1* knockdown. We examined whether ablation of *HDAC1* resulted in compensatory upregulation of any other members of the HDAC family of proteins in hGSCs (Figure 3A). In all cell lines, *HDAC2* and *HDAC3* expression levels were modestly but significantly reduced after *HDAC1* knockdown (Figure 3A). The only HDACs that were significantly upregulated following *HDAC1* loss were *HDAC9* in NHAs and *HDAC10* and *HDAC11* in BT187 (Figure 3A). However, overall our data suggest that unlike the compensation seen in normal neural stem cells (21), *HDAC1* knockdown did not dramatically affect expression of other HDACs in the majority of the cell lines tested.

HDAC1 and HDAC2 are highly homologous and shown to have individual and overlapping regulatory functions for proliferation and cell survival in other tissues (22). To understand whether these paralogues harbor nonredundant functions in hGSCs, we performed knockdown of *HDAC2* in hGSCs to see whether silencing its expression would result in a phenotype comparable to that of *HDAC1* knockdown (Figure 2A). After *HDAC2* knockdown, HDAC1 protein levels were significantly (~1.5- to 2-fold) upregulated as a result of *HDAC2* silencing in both p53-WT and p53-mutant hGSCs (Figure 3, B and C). Unlike *HDAC1* knockdown, we found that knockdown of *HDAC2* did not significantly affect the viability of p53-WT and p53-mutant hGSCs (Figure 3D and Supplemental Figure 4A). Additionally, knockdown of *HDAC2* did not result in increased p53 K382 acetylation, suggesting that p53 activation is a direct result of *HDAC1* silencing. Hence, we demonstrate that HDAC1 harbors unique functional roles in hGSCs that cannot be compensated for by HDAC2, and we identify HDAC1 as the essential class I deacetylase for hGSC proliferation and survival.

### HDAC1 knockdown results in increased histone acetylation and reduces expression of key stemness and cell fate factors.

**Figure 4:**
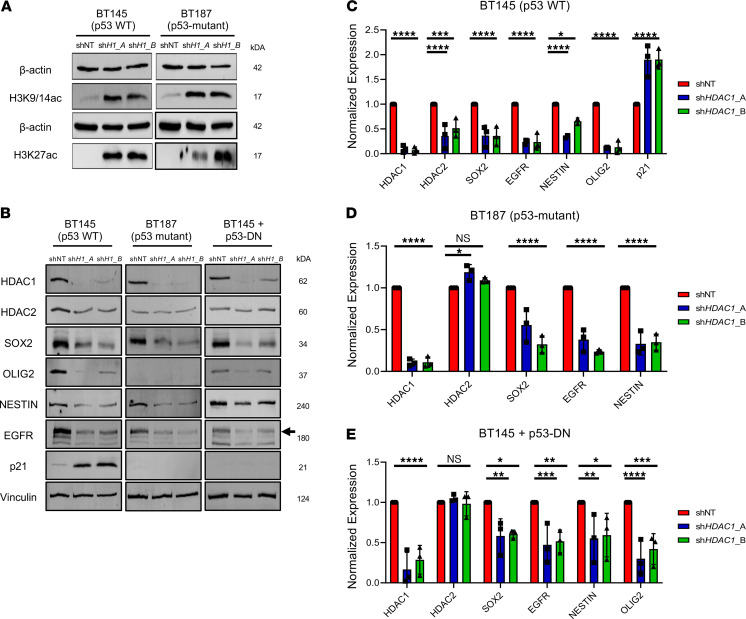
*HDAC1* knockdown reduces expression of key stemness and cell fate factors. (**A**) Immunoblots showing increase in H3K4/19ac and H3K27ac after *HDAC1* silencing in BT145 and BT187 (sh*H1*_A, sh*HDAC1*_A; sh*H1B*, sh*HDAC1*_B) (*n* = 3). (**B**) Representative immunoblots of p53-WT (BT145), p53-mutant (BT187) and p53-WT cells overexpressing p53-DN (BT145 + p53-DN) hGSCs after acute silencing of *HDAC1* probed for various markers (*n* = 3). Black arrow indicates expression of WT EGFR. (**C**–**E**) Quantification of expression of proteins (normalized to Vinculin) after *HDAC1* knockdown from immunoblots using p53-WT (**C**), p53-mutant (**D**), and p53-WT cells overexpressing p53-DN (**E**). For each cell line, the data are compiled from at least 3 independent experiments for each shRNA. Error bars indicate SEM. *P < 0.05, **P < 0.01, ***P < 0.001, ****P < 0.0001. *P* values were determined using the 2-way ANOVA with Tukey’s multiple comparisons test.

To assess the impact of HDAC1 knockdown on global histone acetylation, we analyzed changes in common histone acetylation marks. We first confirmed that knockdown of *HDAC1* in both cell lines led to a dramatic increase in the acetylation of histone H3 at lysines 9, 14, and 27 (H3K9/14 and H3K27). These histone marks are associated with higher activation of gene transcription: H3K9/14ac is highly correlated with active promoters, while the H3K27ac is an active enhancer mark (Figure 4A). Loss of HDAC1 in embryonic stem cells affects stem cell proliferation due to increase in cell cycle inhibitor expression (39). We observed marked upregulation of p21, a direct transcriptional target of p53, in the p53-WT but not p53-mutant cell line (Figure 4, B–D). We then evaluated changes in the expression of several proteins associated with stem cell identity or cell fate in p53-WT (BT145) and p53-mutant (BT187) hGSC lines. Knockdown of *HDAC1* resulted in a significant decrease in the expression of glioma master transcription factors SOX2 and OLIG2, stem cell marker NESTIN, and the receptor tyrosine kinase epidermal growth factor receptor (EGFR; Figure 4, B–D). Overexpression of p53-DN mutant in p53-WT cells also resulted in similar expression changes (Figure 4, B and E). While HDAC2 protein levels did dramatically decrease in the absence of HDAC1 in p53-WT hGSCs (Figure 4C), they remained unchanged in p53-mutant cells, p53-WT cells overexpressing p53-DN, ihNPCs, and NHAs after HDAC1 knockdown (Figure 4, D and E; Supplemental Figure 3D; and Supplemental Figure 4B). Hence, while HDAC1 expression is upregulated in the absence of HDAC2 protein (Figure 3, B and C), HDAC2 does not display any compensatory upregulation after *HDAC1* knockdown in hGSCs (Figure 4, B–E, and Supplemental Figure 4B). Together, these data indicate that knockdown of HDAC1 results in a collapse of the stemness state (irrespective of p53 status) and activation of p53 downstream target genes in surviving p53-WT, but not in p53-mutant, hGSCs (Figure 4B).

### HDAC1 knockdown in GSCs markedly attenuates their ability to form tumors and increases survival of tumor-bearing mice.

**Figure 5:**
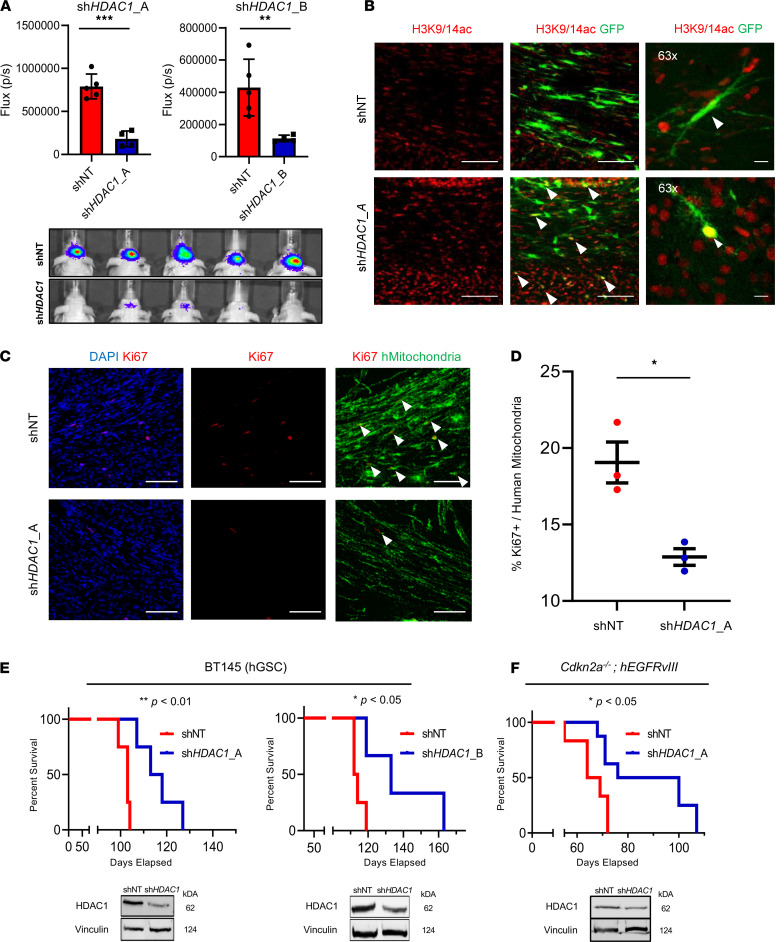
Knockdown of *HDAC1* significantly extends survival in PDX and mouse models of GBM. (**A**) Average photon flux (p/s) measured 7 weeks postinjection through bioluminescence imaging of mice implanted with cells expressing control (shNT) and HDAC1-knockdown (*shHDAC1_A* and *shHDAC1*_B) cells and representative heatmap of bioluminescence intensity between the 2 groups. (**B**) Immunostaining for acetylated histone H3 at lysines 9 and 14 (H3K9/14ac; red) in tumor tissue. Arrowheads indicate GFP-positive cells with H3K9/14a-positive nuclei. (**C**) Immunostaining for Ki67 (red) and human mitochondria (hMitochondria, green) in shNT and sh*HDAC1* BT145 tumor tissue. Arrowheads indicate double-positive (Ki67^+^hMitochondria^+^) nuclei. (**D**) Quantification of human Ki67-positive cells in shNT and sh*HDAC1* BT145 tumors (*n* = 3 per cohort). (**E**) Kaplan-Meier survival analysis of mice implanted intracranially with p53-WT hGSCs (BT145) transduced with *HDAC1* shRNA (sh*HDAC1*_A, *n* = 4; sh*HDAC1*_B, *n* = 3) or nontarget shRNA (shNT; *n* = 4 in both studies). (**F**) Kaplan-Meier survival analysis of mice implanted intracranially with murine GSCs (*Cdkn2a^–/–^* *hEGFRvIII*) transduced with *HDAC1* shRNA (sh*HDAC1*_A, *n* = 5) or shNT (*n* = 4). Inset below shows immunoblots confirming *HDAC1*-knockdown in the implanted GSCs. Error bars indicate SEM. *P < 0.05, **P < 0.01, ***P < 0.001. Original magnification, 20× and 63×; scale bars, 100 μm. *P* values were calculated using unpaired 2-tailed *t* test and Kaplan-Meier method with the Mantel-Cox log-rank test. See also Supplemental Figure 5.

We next investigated whether loss of HDAC1 affected the tumor-forming potential of p53-WT hGSCs. We orthotopically transplanted hGSCs (BT145) transduced with a luciferase reporter and either an shNT or 2 *HDAC1*-targeting shRNAs in immunocompromised mice. Using bioluminescence imaging, we compared the differences in the tumor growth rate in shNT and sh*HDAC1* tumors at an early time point after hGSC engraftment (7 weeks postinjection; Figure 5A). *HDAC1* knockdown resulted in a significant lag in tumor growth with both shRNAs, which was maintained throughout the study (Figure 5A and Supplemental Figure 5, A and B). We confirmed that *HDAC1* silencing was maintained in sh*HDAC1* tumors (Supplemental Figure 5, C and D). In line with our in vitro phenotypes (Figure 4B), we also confirmed that sh*HDAC1* tumors expressed very low levels of OLIG2 — a master transcription regulator that has previously been shown to be critical for the tumor-propagating potential of p53-WT GSCs (Supplemental Figure 5C and refs. 36, 40). Consistent with low HDAC1 expression, the engrafted sh*HDAC1* cells exhibited an increase in H3K9/14ac relative to shNT controls (Figure 5B). As shown in Figure 5, C and D, sh*HDAC1* tumors had a significant reduction in the proportion of proliferating cells compared with the controls. Knockdown of *HDAC1* alone not only delayed tumor growth but also resulted in significantly extended overall survival in a PDX model of GBM (BT145; Figure 5E) and in a murine model of human glioma (Figure 5F).

### HDAC1 knockdown affects expression of genes involved in apoptosis, the cell cycle, and cellular communication and migration in p53-WT hGSCs.

**Figure 6:**
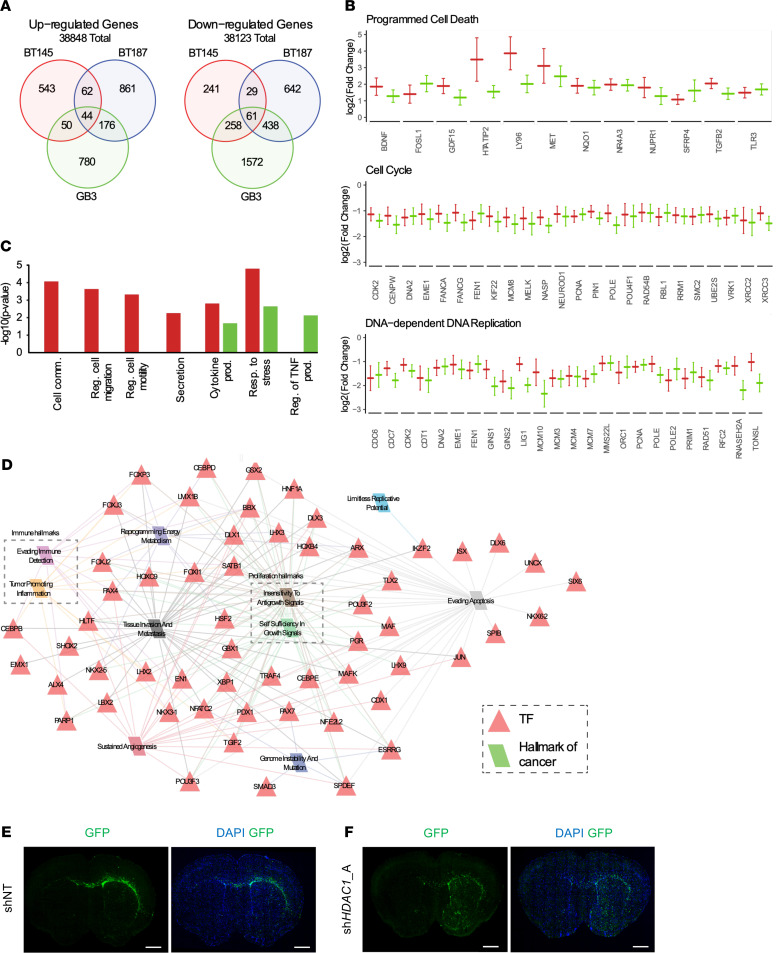
*HDAC1* knockdown upregulates cell migration programs and results in more invasive tumors. (**A**) Venn diagram illustrating the overlap between the significantly up- and downregulated genes between GSC cell lines after sh*HDAC1* knockdown. (**B**) Log_2_ fold change of gene expression after sh*HDAC1* knockdown in p53-WT hGSCs BT145 (red) and GB3 (green) hGSCs (adjusted *P* ≤ 0.05). (**C**) Negative log_10_ *P* value for functional enrichment of relevant Gene Ontology Biological Process (GO BP) terms for genes with significantly increased gene expression after sh*HDAC1* knockdown in BT145 (red) and/or GB3 (green) tumorigenic cell lines. (**D**) BT145 and GB3 specific regulatory network for upregulated genes following *HDAC1* knockdown. Red triangles are transcription factor regulators and parallelograms are hallmarks of cancer. Edges indicate association between the target genes of the regulator and a hallmark of cancer and are colored according to its corresponding hallmark. For each cell line, the data are compiled from 3 independent experiments. (**E** and **F**) Stitched whole-brain images of DAPI (blue) and GFP-positive engrafted tumor cells (green) in (**E**) shNT and (**F**) sh*HDAC1* BT145 brain tissue 7 weeks postengraftment. GFP expression reveals *HDAC1*-deficient tumors are more invasive than control shNT tumors. Original magnification, 10×; scale bars, 1 mm. See also Supplemental Figure 6.

In our RNA-Seq analysis, we identified 2516 differentially upregulated and 3241 differentially downregulated genes across the 3 hGSC lines analyzed (Figure 6A). Although p53-WT cell lines had a similar phenotypic response to loss of HDAC1, each cell line appeared to have substantially different responses to HDAC1 knockdown albeit with some overlapping genes, independent of p53 status. This may be partly explained by the distinct genotypes/driver mutations present in each cell line (e.g., p53-WT lines BT145 and GB3 are *EGFR* and *PDGFRA* driven, respectively), such that *HDAC1* ablation may impart similar phenotypes through regulation of different sets of genes. We performed functional enrichment analyses in p53-WT cells (BT145 and GB3) compared with p53-mutant cells (BT187). Common genes significantly upregulated in p53-WT cell lines were associated with programmed cell death while genes associated with cell cycle and DNA-dependent DNA replication were significantly downregulated (Figure 6B). These data are consistent with our in vitro results following *HDAC1* silencing (Figure 2, A–E, and Figure 4B). Interestingly, in p53-WT hGSCs we also found significant enrichment of genes associated with cellular communication, cellular migration, cytokine production, and responses to stress (Figure 6, C and D).

### HDAC1 knockdown results in aberrant differentiation and invasion of p53-WT hGSCs.

Prolonged treatment with pan-HDACi has previously been shown to induce differentiation in hGSCs (41). Regulatory network analysis identified 61 transcription factors associated with the regulation of genes upregulated after *HDAC1* knockdown (Figure 6D). Interestingly, upregulated genes include neuronal differentiation transcription factors such as *DLX1*and *DLX*6 (42–44), oligodendrocyte differentiation transcription factors such as *NKX6-2* (45) and *EMX1* (46), and transcription factors expressed in developing and mature astrocytes such as *NFATC2*, *CEBPB*, *CEBPE*, and *POU3F2* (47).

Considering that knockdown of *HDAC1* led to significant upregulation of expression of transcription factors that promote tissue invasion (48–50) (e.g., *NFATC2* and *CEBPB*; Figure 6D), we questioned whether sh*HDAC1* tumors exhibited a different growth pattern in vivo. While there was a marked difference in tumor burden across the 2 groups 7 weeks postimplantation, extensive infiltration of GFP-positive sh*HDAC1* tumor cells was seen throughout the brain parenchyma compared with shNT tumor cells — demonstrating that HDAC1-deficient tumors displayed a more invasive phenotype in vivo (Figure 6, E and F).

Ablation of *HDAC1* expression did not result in significant shifts between different cellular states described by Neftel et al., (51), although some trends were evident: BT145 cells adopted a more astrocyte-like state, and GB3 cells adopted a more hypoxia-dependent mesenchymal-like (MES2) state, while p53-mutant BT187 cells tended to shift from a hypoxia-independent (MES1) to a hypoxia-dependent MES2 state (Supplemental Figure 6). Our results suggest that *HDAC1* knockdown results in transcriptional changes that not only alter cell survival and death programs but also alter cell invasion in hGSCs in a p53-dependent manner.

### Genetic ablation of HDAC1 results in increased STAT3 activity in p53-WT hGSCs.

**Figure 7:**
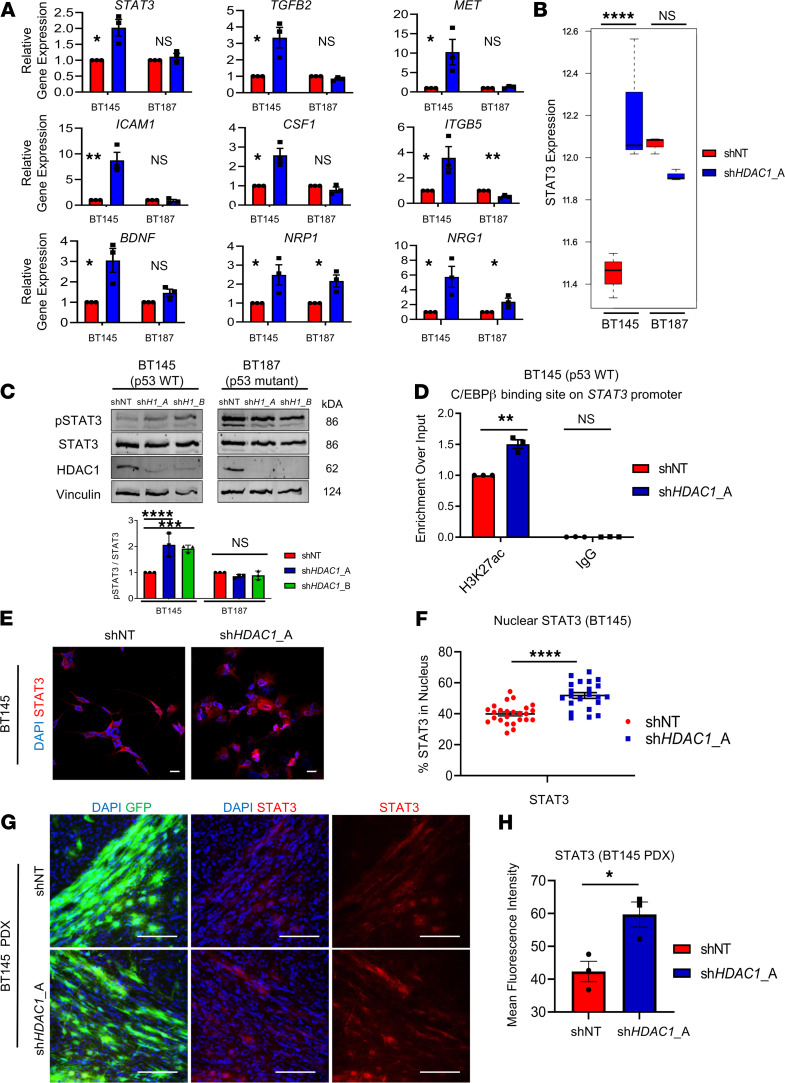
*HDAC1* knockdown results in increased STAT3 signaling in p53-WT hGSCs. (**A**) RT-qPCR for genes involved in cellular invasion or survival in BT145 (p53-WT) and BT187 (p53-mutant) hGSCs (*n* = 3 per target). (**B**) RNA-Seq analysis for *STAT3* expression in BT145 and BT187 after HDAC1 knockdown. The box plots depict the minimum and maximum values (whiskers), the upper and lower quartiles, and the median. The length of the box represents the interquartile range. (**C**) Lysates were collected from BT145 and BT187 after acute silencing of *HDAC1* (sh*H1*_A, sh*HDAC1*_A; sh*H1*_B, sh*HDAC1*_B). Immunoblots were probed with antibodies for phosphorylated STAT3 (Tyr705), STAT3, HDAC1, and Vinculin. Bar graph below shows quantification of the normalized ratio of p-STAT3 over total STAT3 protein after *HDAC1* knockdown in BT145 and BT187 hGSCs (*n* = 3). (**D**) Chromatin immunoprecipitation assay for H3K27ac deposition in the C/EBPβ binding site on the *STAT3* promoter in BT145 (*n* = 3). (**E**) Immunocytochemistry staining for STAT3 in BT145 after acute *HDAC1* knockdown. (**F**) Quantification of immunocytochemistry experiments showing significantly increased nuclear localization of STAT3 after HDAC1 knockdown in BT145. Graph shows values from individual experimental values from multiple experiments (*n* = 4). (**G**) Immunofluorescence staining for STAT3 in BT145 PDX tumor tissue 7 weeks postengraftment in *HDAC1*-silenced tumors relative to controls. (**H**) Quantification of mean pixel intensity for STAT3 staining in BT145 shNT and sh*HDAC1* PDX tumors. Graph shows average values from 3 independent animals per experimental condition. Error bars indicate SEM. *P < 0.05, **P < 0.01, ***P < 0.001, ****P < 0.0001. For each cell line, the data are compiled from at least 3 independent experiments. Original magnification, 20×; scale bars, 2 μm and 100 μm. *P* values were calculated using unpaired 2-tailed *t* test or 2-way ANOVA with Tukey’s multiple comparisons test. See also Supplemental Figure 7.

We validated upregulation of genes involved in regulating cancer cell invasion, migration, and survival upon *HDAC1* knockdown using quantitative reverse transcription PCR (RT-qPCR) in p53-WT (BT145) and p53-mutant (BT187) hGSCs (Figure 7A). We found that expression of *STAT3*, *TGFB2*, *MET*, *ICAM1*, *CSF1*, *ITGB5*, *BDNF*, *NRP1*, and *NRG5* was significantly induced after *HDAC1* knockdown almost exclusively in p53-WT but not p53-mutant hGSCs (Figure 7A). Out of the 9 genes we analyzed, 5 have been shown to be direct targets of or are modulated by p53: *TGFB2*, *STAT3*, *MET*, *ICAM1*, and *CSF1* (52–56). Several of these genes (*BDNF*, *TGFB2*, *MET*) are also direct target genes of transcription factors that are upregulated after *HDAC* knockdown in p53-WT hGSCs (Figure 6D).

We were particularly intrigued to find that *STAT3* — an important oncogenic driver and established master regulator of mesenchymal transformation in GBM (57, 58) — was significantly induced after *HDAC1* knockdown uniquely in p53-WT hGSCs (Figure 7, A and B). Moreover, in our transcriptional regulatory network analysis (Figure 6D), we found that *CEBPB* — regulator of *STAT3* expression in GBM (57) — was significantly upregulated in p53-WT hGSCs. Given the intrinsic phenotypic plasticity of glioma cells, we sought to determine whether the STAT3 signaling axis could be a potential compensatory mechanism that is adopted by surviving *HDAC1*-deficient hGSCs. Downstream STAT3 enrichment analysis from our RNA-Seq data sets revealed that several STAT3 target genes were significantly differentially regulated in p53-WT hGSCs (Supplemental Table 3). In addition to increased STAT3 mRNA expression, the phosphorylation of STAT3 at tyrosine 705 (p-STAT3), a transcriptionally active form of the protein, increased significantly following *HDAC1* silencing in p53-WT hGSCs (Figure 7C). The levels of p-STAT3 are already elevated in p53-mutant hGSCs and remained unchanged following *HDAC1* knockdown (Figure 7C), and this phenotype could be reproduced when we overexpressed a p53-DN in p53-WT hGSCs (Supplemental Figure 7). Chromatin immunoprecipitation assay supported that *HDAC1* knockdown resulted in increased H3K27ac deposition in the C/EBPβ binding site on the *STAT3* promoter region relative to control p53-WT cells (Figure 7D). In agreement with increased STAT3 phosphorylation, we observed increased nuclear localization of STAT3 in p53-WT cells after *HDAC1* knockdown (*P* < 0.0001; Figure 7, E and F). Furthermore, we also showed that STAT3 expression was significantly elevated in sh*HDAC1* p53-WT PDX tumors relative to control tumors (*P* < 0.05; Figure 7, G and H).

## Discussion

Considering the role of HDACs in oncogenesis, there has been an increased interest in testing HDACi for several malignancies, including both adult and pediatric gliomas (7, 59). However, almost all HDACi used in clinical trials for primary and recurrent GBM to date are unable to provide significant therapeutic benefit to patients (60). These HDACi (e.g., TSA, vorinostat, SAHA, panobinostat) are broad-spectrum and have poor pharmacokinetic profiles as well as a narrow therapeutic index (4–8). Isoform specificity for HDACi is important given that not all HDAC enzymes are equally expressed in GBM (12). Most of our knowledge on the presumed roles of HDACs in gliomas is largely based on preclinical studies with pan-HDACi, which preclude a true understanding of the requirement and role of individual HDACs in these tumors, especially within the therapy-resistant cancer stem cells (24, 61, 62). To this end, we specifically investigated the functional importance of HDAC1 in GSCs, an HDAC isoform whose expression increases with brain tumor grade and is correlated with decreased survival (12, 28) (Figure 1). Although several CRISPR/Cas9 screens conducted on hGSCs did not identify *HDAC1* as an essential fitness gene governing hGSC growth and survival (63, 64), recent studies corroborate our findings that HDAC1 is an attractive therapeutic target for GBM (23, 24). Bastola et al. identified HDACi through a large-scale screen of small molecules that demonstrated increased efficacy in targeting glioma cells within the tumor core versus the invasive edge (24). They also identified HDAC1 as a regulator of CD109 expression, a marker of aggressive mesenchymal GBM cells. Song et al. focused on the role of NFAT2 and demonstrated that NFAT2 binds to the *HDAC1* promoter and regulates its expression to promote mesenchymal transformation of hGSCs (23). Here, we demonstrate that genetic silencing of *HDAC1* alone attenuates the tumorigenic and stemness potential of GSCs in a p53-dependent manner and substantially extends survival in PDX and mouse models of GBM in vivo. Our data provide a rationale for the development of isoform-specific HDACi for the treatment of GBM.

Although HDAC1 and HDAC2 have been shown to be functionally redundant in many cellular contexts because of their high sequence homology, several studies have demonstrated that this redundancy is not observed during early embryogenesis and central nervous system development (21, 65, 66). For instance, targeted deletion of *Hdac1* in mice results in embryonic lethality due to severe proliferation and differentiation defects during development (67). On the contrary, in the developing brain, HDAC2 was identified as being both essential and sufficient to ensure normal brain development and survival in the absence of HDAC1 in *Nestin*-Cre–transgenic mice (21). Here, we show that HDAC1 is required for the viability and proliferative potential of p53-WT GSCs, while p53-mutant GSCs, normal neural progenitor cells, and astrocytes were significantly but more modestly affected by *HDAC1* silencing (Figure 2A). Given that we did not observe increased HDAC2 activity following *HDAC1* knockdown, the severe phenotype exhibited by HDAC1-deficient GSCs reveals that HDAC1 and HDAC2 harbor previously underappreciated, nonredundant functions in GBM. This is further highlighted by our observations that HDAC2 silencing did not affect the viability of p53-WT and p53-mutant hGSCs and that HDAC1 displayed significant compensatory upregulation in these cells when *HDAC2* expression was silenced (Figure 3, B and C). With respect to the impact of *HDAC1* silencing in normal cells (NHAs, ihNPCs), our results support previous studies that have shown that deletion of *Hdac1* in mature astrocytes does not result in cellular catastrophe and that HDAC2 is the essential class I deacetylase in neural progenitor cells in absence of its paralogue (21, 68). We did see a marked increase in *HDAC10* and *HDAC11* in p53-mutant GSC lines and *HDAC9* in NHAs, so it is likely that these HDACs play a compensatory role in astrocytes and p53-mutant lines.

Our findings suggest that HDAC1 function may be dispensable or irrelevant in a context where p53 is mutated in GSCs. Unlike p53-WT GSCs, we did not observe p53-mediated cell growth arrest and apoptosis in p53-mutant cells after acute *HDAC1* silencing (Figure 2). Despite this, we found that *HDAC1* knockdown still resulted in an average 30%–40% loss in cell viability in p53-mutant hGSCs. This phenomenon might be explained by previous studies that have shown that the presence of functional p53 can dictate the fate of various cancer cell lines in response to HDACi, wherein p53-WT cells preferentially undergo apoptosis while p53-deficient or p53-mutant cells undergo autophagy (69, 70). Whether p53 status dictates the induction of autophagy over apoptosis in the absence of specific HDAC isoforms (such as HDAC1) in hGSCs remains an important question to be addressed in future studies.

**Figure 8:**
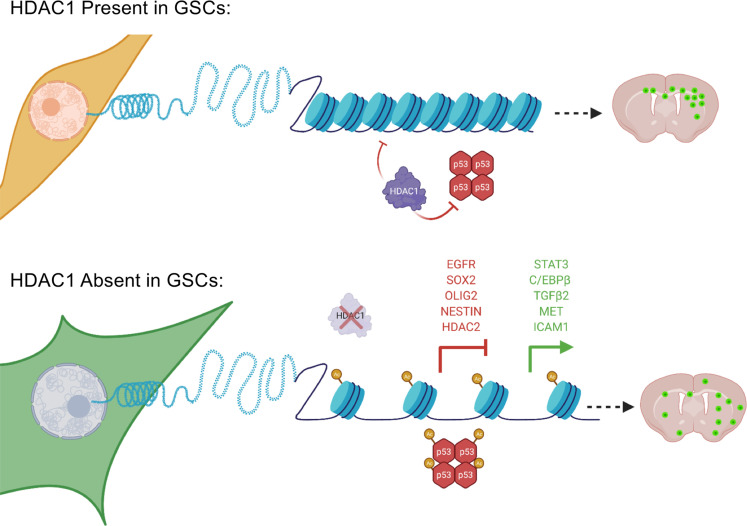
Proposed model: consequences of *HDAC1* silencing in p53-WT hGSCs. Summary of the cellular and molecular effects of *HDAC1* loss in p53-WT hGSCs. Absence of HDAC1 results in increased histone acetylation and restoration of p53 activation and stability. These changes are accompanied by marked changes in gene expression, wherein genes involved in maintaining stemness are downregulated while genes involved in promoting differentiation and cellular migration and communication are upregulated. In vitro, these cells fail to proliferate and die; however, when transplanted in vivo these cells form slower growing but more invasive tumors. STAT3 activity, which is known to drive aggressive phenotypes in GBM, is upregulated after HDAC1 loss and may be a potential druggable compensatory pathway that may be targeted in combination with more selective HDAC1i.

Our results also yield new insights into the previously described molecular mechanism underlying the oppositional relationship between OLIG2 and p53 present in both neural progenitors and malignant glioma (36). Olig2 has been shown to directly repress p21 and oppose p53 functions by suppressing its acetylation (thereby activation) and transcriptional functions (36, 71). Here we show that *HDAC1* knockdown in p53-WT hGSCs results in downregulation of OLIG2, induction of p21, and increased p53 stabilization and acetylation (Figure 3G, Figure 4B, and Figure 8) as previously hypothesized by Meijer et al. (72). Additionally, our observations are consistent with reports demonstrating that deacetylation of p53 is primarily mediated by HDAC1 and not HDAC2 in various nonglioma cell lines (e.g., 293T, 3T3 cells; refs. 25, 26) and is indicative of the reversal of OLIG2-mediated suppression of p53 DNA targeting (36). Acetylation has been shown to be important to promote increased protein stability and transcriptional functions of p53; however, we do not exclude that there are several other possible mechanisms that can affect the regulation of p53 gene expression in the absence of HDAC1 (73). Previous studies have shown that certain RNA-binding proteins can control the kinetics of *TP53* mRNA translation (74). However, since we did not observe increased transcription of *TP53* mRNA in p53-WT hGSCs after *HDAC1* knockdown (Supplemental Figure 3G), the observed increase in p53 target gene expression (e.g., p21) might be due to either increased stability of p53 protein and/or transcriptional activity of p53. In addition to acetylation, other posttranslational modifications such as phosphorylation and methylation can also promote p53 stability by enhancing interactions with acetyltransferases and blocking MDM2 association (75, 76).

Knockdown of *HDAC1* — a single HDAC isoform — is sufficient to prolong survival in vivo (Figure 5, E and F). Intriguingly, although *HDAC1*-silenced p53-WT hGSCs fail to proliferate and die in vitro, these cells can form tumors in immunocompromised mice — albeit at a slower rate compared with controls (Figure 5A and Supplemental Figure 5, A and B). This strongly suggests that standard GSC in vitro culture conditions may lack the appropriate soluble factors and cellular interactions that are required for these cells to thrive in the absence of HDAC1 activity or that a persister population of cells survives *HDAC1* ablation in vivo (77). Our transcriptional regulatory network analysis (Figure 6D) revealed that HDAC1 silencing in p53-WT hGSCs resulted in the upregulation of multiple transcription factors that are associated with several hallmarks of cancer (e.g., tumor-promoting inflammation, evasion of apoptosis, tissue invasion, and metastasis), which promote tumor growth and survival. The observation that sh*HDAC1* tumors are significantly more invasive at early stages of tumorigenesis thereby prompted us to investigate potential alternative mechanisms that are adopted by these cells to grow in the absence of HDAC1 (Figure 6, E and F). Indeed, we found that ablation of *HDAC1* expression resulted in marked upregulation of a vast array of genes that have previously been implicated in promoting tumor cell survival, invasion, and metastasis in gliomas and other cancers (e.g., *TGFB2*, *ITGB5*, *CSF1*, *NRG1*, *NRP1*, *ICAM1*, *MET*; Figure 7A, Figure 8, and refs. 78–85).

We identified STAT3 signaling axis as a promising and druggable compensatory pathway that is upregulated after HDAC1 loss in p53-WT hGSCs. STAT3 is an established master regulator of the mesenchymal gene expression signature in GBM, which imparts a highly aggressive, treatment-resistant phenotype that predicts poor clinical outcome (57, 86, 87). While we did not observe any evident shift toward the mesenchymal cell state in our cells (Supplemental Figure 6), STAT3 is known to promote tumor survival and proliferation and is a major driver of glioma cell migration and invasion (88). Moreover, HDAC1 has previously been implicated in regulating STAT3 nucleocytoplasmic partitioning and activity, wherein HDAC1 expression was shown to reduce nuclear accumulation of STAT3 in commonly used cancer and nontumorigenic cell lines (89–91). Indeed, we observed nuclear translocation of STAT3 in p53-WT GSCs after HDAC1 knockdown (Figure 7E).

We found that in p53-mutant cells and p53-WT cells overexpressing a p53 mutant, which were only modestly affected by HDAC1 silencing, p-STAT3 levels were already elevated at baseline and did not change after HDAC1 loss — this is in line with previous studies that have shown cancer cell lines that harbor p53 mutations or deletions express constitutively active STAT3 (Figure 7C, Supplemental Figure 7, and refs. 56, 92, 93). These data highlight the importance of the HDAC1/STAT3 signaling axis, which has practical overtones for combination therapy in p53-WT GBMs. As seen in our RNA-Seq data, knockdown of *HDAC1* results in distinct changes in every cell line (p53-WT and mutant). Hence, future studies are required to identify additional genetic markers beyond p53 that predict responsiveness to HDAC1 inhibition. Considering the plastic nature of glioma cells and transitions between cell states (51), relying on molecular subtypes to identify HDACi-responsive tumors might not be a reliable approach.

As seen from clinical trials with class I–specific HDACi such as romidepsin (6), it is unlikely that a monotherapeutic strategy with more isoform-selective HDACi would result in a durable response in patients. Based on our data, we propose that combination therapies with brain-penetrant STAT3 inhibitors is a promising treatment strategy for p53-WT GBM tumors. Although preclinical studies with several HDACi have been shown to suppress growth in murine PDX models (24, 61), these inhibitors are either toxic at human-equivalent doses and/or have high plasma protein binding (>96% for AR-42; ref. 94), which will hinder translating these findings to the clinic. A putative brain-available STAT3 inhibitor, WP1066, is currently under clinical trial (NCT01904123). Future combination studies with isoform-specific HDACi and STAT3 inhibitors that incorporate pharmacokinetic and pharmacodynamic analysis in multiple PDX models will allow translation of these findings to the clinic.

In summary, our data highlighted an unmet need for isoform-specific HDAC inhibitors in GBM and identified a potential druggable compensatory mechanism that can guide future combination studies.

## Methods

### Primary cell culture.

Patient-derived glioma stem cell lines (hGSCs; GB3, GB82, GB71, and GB84) were established from resected primary GBM tumor tissue at Barrow Neurological Institute (BNI). BT145, BT70, BT187, and BT286 hGSCs were obtained from Keith Ligon’s laboratory at the Dana-Farber Cancer Institute, Boston, Massachusetts, USA. Finally, 211 and 252 hGSCs were obtained from Harley Kornblum’s laboratory at University of California, Los Angeles, Los Angeles, California, USA. All human GSCs were cultured as described previously (36, 71). NHAs were purchased from Thermo Fisher Scientific. Human neural progenitor cells (hNPCs) were derived from iPSCs from control subjects at the BNI (ihNPCs) (provided by Rita Sattler’s laboratory). ihNPCs, hGSCs, and murine GSCs were cultured as spheres on non–tissue culture–treated 10 cm plates or as adherent cultures on laminin on tissue culture–treated 10 cm plates (Thermo Fisher Scientific). ihNPCs and hGSCs were grown in DMEM/F12 media, supplemented with B27 and N-2 (Invitrogen, Thermo Fisher Scientific) in the presence of 20 ng/mL epidermal growth factor and basic fibroblast growth factor (MilliporeSigma). Primary human astrocytes (NHA; ScienCell) were grown in Astrocyte Media (5% FBS, Astrocyte Growth Supplement, 1% pen-strep) on poly-l-lysine–coated plates according to manufacturer recommendations.

### Lentivirus production.

Lentiviruses were generated in HEK293FT cells (Thermo Fisher Scientific, R70007) through Polyfect-mediated (QIAGEN) transfection with fourth-generation packaging plasmids coding for HDMH.Hgpm2, TAT, REV, HDM.G, and lentiviral transfer vector (pGFP-C-shLenti). The *HDAC1*-targeting shRNA lentiviral vectors were obtained from Origene (sh*HDAC1*_A: 5′-GTCCAAAGTAATGGAGATGTTCCAGCCTA-3′ and sh*HDAC1*_B: 5′-ATTTGCTGCTCAACTATGGTCTCTACCGA-3′). The *HDAC2*-targeting shRNA lentiviral vector was obtained from Horizon Discovery (sh*HDAC2*: 5′-TCATGAACAGCATCTTCTG-3′). As a negative control we used a nontarget shRNA sequence (shNT: 5′-CAACAAGATGAAGAGCACCAA-3′), which does not target any human genes. Retroviruses were generated in HEK293FT cells by Polyfect-mediated transfection with packaging plasmids encoding VSVG and gag-pol and a retroviral transfer vector (pWZL) encoding the gene of interest (p53-DN; ref. 36).

### Cell viability assays.

GSCs, hNPCs, and NHAs transduced with either shNT or sh*HDAC1* shRNAs at an MOI of 3 were selected with 2 μg/mL puromycin 72 hours posttransduction. Control or *HDAC1*-knockdown GSCs and NPCs were seeded in laminin-coated, tissue culture–treated, 96-well plates (clear bottom, white plate; Corning) at a density of 1000–2000 cells per well (cell line dependent) in GSC media (described in *Primary cell culture*). Transduced NHAs were seeded using their normal growth conditions without laminin (10% FBS in DMEM). Seeded cells were grown for 7 days at which point cell viability was measured and quantified. All cell viability measurements were performed using the CellTiter-Glo Luminescent Cell Viability Assay (Promega) following the manufacturer’s instructions. All cell viability results represent the mean of at least 2 biological replicates, each containing 3 technical replicates.

### Western blotting.

Cellular protein from cultured cells was homogenized in RIPA lysis buffer containing protease and phosphatase inhibitors (Thermo Fisher Scientific), rotated at 4°C for 20 minutes, and then centrifuged at 25,200*g* for 10 minutes at 4°C. Protein concentration from whole-cell extracts was determined using the Bradford Protein Assay (Thermo Fisher Scientific). Equal amounts of protein (10–40 μg/lane) were loaded onto a 7.5%, 10%, or 12.5% SDS-PAGE gel and transferred to a polyvinylidene fluoride membrane (MilliporeSigma).

Membranes were blocked with 5% nonfat milk for 1 hour at room temperature and incubated overnight with primary antibody at 4°C. Primary antibodies used in this study were mouse anti-HDAC1 (1:1000; Cell Signaling Technologies, 5356), rabbit anti-HDAC1 (1:1000, Cell Signaling Technologies, 2062), rabbit anti-HDAC2 (1:1000; Cell Signaling Technologies, 2540), mouse anti-HDAC3 (1:1000, Cell Signaling Technologies, 3949), rabbit anti-SOX2 (1:1000; Cell Signaling Technologies, 3579), mouse anti-human Nestin (1:1000; Novus Biologicals, 10C2), rabbit anti-OLIG2 (1:1000, gift from the Stiles lab, Dana-Farber Cancer Institute), rabbit anti-p21 (1:500; Abcam, ab109520), rabbit anti-EGFR (1:10,000; Abcam, ab52894), mouse anti-STAT3 (1:1000, Cell Signaling Technologies, 9139), rabbit anti–p-STAT3 Tyr705 (1:500, Cell Signaling Technologies, 9145), mouse anti-p53 (1:1000, Cell Signaling Technologies, 2524), rabbit anti-acetyl p53 K382 (1:500, Cell Signaling Technologies, 2570), rabbit anti-acetyl p53 K373 (1:5000, Abcam, 62376), rabbit anti-H3K27ac (2 μg/mL, Abcam, ab4729), rabbit anti-H3K9/14ac (1:1000, Cell Signaling Technologies, 9677), mouse anti–β-actin (1:1000, Bio-Rad, MCA5775GA), and mouse anti-Vinculin (1:10,000, MilliporeSigma, 05-386). Membranes were probed with fluorophore-conjugated anti-mouse or anti-rabbit secondary antibodies (1:10,000; Thermo Fisher Scientific). Western blots were developed using the LI-COR Odyssey CLx imaging system (LI-COR Inc.) and quantitated using the Image Studio Lite software. All Western blots are representative images from a minimum of 3 biological replicates.

### Immunocytochemistry.

Cells were grown as adherent cultures on laminin-coated glass coverslips (Thermo Fisher Scientific) in GSC media. Twenty-four hours after plating,the cells were transduced with nontarget control or HDAC1-targeting shRNAs at an MOI of 3. Seventy-two hours posttransduction, puromycin was added at a concentration of 2 μg/mL for 72 hours to select for successfully transduced cells. After selection cells were fixed with 4% paraformaldehyde for 13 minutes at room temperature. Cells were washed with PBS and subsequently permeabilized and blocked with 5% normal goat serum (MilliporeSigma) and 0.2% Triton X-100 in PBS (blocking solution) for 30 minutes at room temperature. The cells were incubated with primary antibodies overnight at 4°C in blocking solution. Primary antibodies used in this study included rabbit anti-Ki67 (1:1000; Abcam, 15580), rabbit anti–cleaved caspase-3 (1:300; Cell Signaling Technologies, 9661), and mouse anti-STAT3 (1:100; Cell Signaling Technologies, 9139). The following day, the cells were washed with PBS 3 times, then incubated with fluorophore-conjugated secondary antibodies at 1:1000 dilutions (Alexa Fluor 568 goat anti-mouse, Abcam, ab175473; Alexa Fluor 568 goat anti-rabbit, Abcam, ab175471) for 1 hour at room temperature, and finally washed in PBS 3 more times. Cells were mounted onto SuperFrost Plus microscope slides using Fluoroshield Mounting Medium containing DAPI (Abcam). Images were acquired using a confocal microscope (Leica Microsystems; TCS SP5) operated with LAS software. The fractions of Ki67- and cleaved caspase-3–positive cells were counted from 5 independent images from each condition. The average and standard deviation were calculated from 3 biological replicates for all control and *HDAC1*-knockdown (shRNAs A and B) experiments.

### RNA-Seq analysis.

GSCs, hNPCs, and NHAs transduced with either shNT or sh*HDAC1* shRNAs at an MOI of 3 were selected with 2 μg/mL puromycin 72 hours posttransduction. Then, 72 hours postselection, total RNA was extracted from cells using the PureLink RNA Mini Kit (Ambion) in accordance to the manufacturer’s instructions. Purified RNA was sent to NovoGene, where the RNA was turned into an RNA-Seq library using the standard Illumina TruSeq method, and sequenced on an Illumina NextSeq 2500 sequencer. FASTQ files were aligned to the genome using STAR (95) and genome build GRCh38 V2.7.1a, counts were tabulated using htseq-count (96), and DESeq2 (97) was used for subsequent differential gene expression analysis. Quality of the sequencing was determined using FastQC, alignment quality was determined by requiring at least 18 million uniquely mapping reads per sample from STAR, and replicate quality was determined through principal component analysis in DESeq2. Differential expression between sh*HDAC1* and shNT was computed in DESeq2, and differentially expressed genes were required to have a log_2_ fold change ≥ 1 and Benjamini-Hochberg–adjusted *P* ≤ 0.05. Eigengenes for the Neftel et al. 2019 developmental subtypes (51) were computed from the genes in Supplemental Figure 6 using the module Eigengenes function in the WGCNA package in R (98). The data discussed in this publication have been deposited in NCBI’s Gene Expression Omnibus (GEO) and are accessible through GEO series accession number GSE179882 (https://www.ncbi.nlm.nih.gov/geo/query/acc.cgi?acc=GSE179882).

### Regulatory network inference.

Genes found to be differentially expressed in BT145, GB3, and BT187 cells were independently analyzed for significant enrichment of transcription factor (TF) binding sites in the promoters (±5 kbp from the transcription start sites of genes; ref. 99) using the TF targets python package (https://github.com/cplaisier/TF_targets). Significantly enriched TFs (*P* ≤ 0.05) that were found to be regulators in both BT145 and GB3 but not BT187 were selected for further analyses. The BT145- and GB3-specific TF regulator target genes (with binding sites in their promoters) were tested for functional enrichment with GO BP terms using the enrichr module from the gseapy package on pypi. Significant functional enrichment was determined by a *P* less than or equal to 0.01. The significantly enriched GO BP terms were then associated with hallmarks of cancer using semantic similarity (99–101). Networks were visualized using Cytoscape (102).

### Orthotopic xenograft studies.

*Foxn1^nu^* nude 5- to 6-week-old male mice (The Jackson Laboratory) were used for in vivo orthotopic transplantation of luciferized BT145 (male) cells transduced with either shNT or sh*HDAC1*_A lentivirus. Nude mice were anesthetized using gaseous isoflurane and immobilized on a Leica stereotaxic instrument (39477001, Leica Microsystems). Following an incision at the midline, a fine hole was drilled 2.5 mm lateral to the bregma. Using a 33-gauge needle syringe (700 series, Hamilton), 2 μL of dissociated viable cells (at a density of 50,000 cells/μL) were injected 2 mm deep from the surface of the skull slowly at a constant rate of 1 μL per minute for 2 minutes. The needle was left for 1 additional minute to prevent reflux of the injected cells and was then slowly removed. The incision was closed with surgical staples. All mice were observed daily and were sacrificed upon the onset of severe neurological symptoms and more than 10% body weight loss. Survival data were plotted and analyzed using GraphPad Prism 8 (GraphPad Software).

### Immunofluorescence.

Immunofluorescence was performed on free-floating PDX brain tissue sections (40 μm sections). Sections were washed in 0.1 M phosphate buffer (PB) 6 times, followed (if required) by antigen retrieval in 10 mM citrate buffer (pH 6.0) at 85°C for 30 minutes on a hot plate. Tissue sections were permeabilized and blocked with 10% goat serum and 0.4% Triton X-100 in 0.1 M PB for 2 hours at room temperature. To prevent unspecific staining, sections were further incubated with goat anti–mouse IgG and goat anti–rabbit IgG (catalog numbers 115-007-003 and 111-005-003, Jackson ImmunoResearch Laboratories Inc.; 1:50) in 0.1 M PB and 0.4% Triton X-100 for 30 minutes at room temperature. Sections were then washed 3 times in 0.1 M PB and then incubated in primary antibodies diluted in 2% goat serum and 0.4% Triton X-100 in 0.1 M PB overnight at 4°C. Following washes in 0.1 M PB, incubation with secondary antibodies (Invitrogen; Thermo Fisher Scientific) was performed for 2 hours at 4°C in 2% goat serum and 0.4% Triton X-100 in 0.1 M PB. Nuclear counterstaining was achieved with DAPI (0.5 μg/mL). The following primary antibodies were used in this study: rabbit anti-Ki67 (1:150, Abcam), anti-human mitochondria (1:200, MilliporeSigma, 133-1), mouse anti-OLIG2 (1:400, MilliporeSigma, 211F1.1), rabbit anti-HDAC1 (1:500, Abcam, 109411), rabbit anti-H3K9/14ac (1:1000, Cell Signaling Technologies, 9677), and rabbit anti-STAT3 (1:500, Cell Signaling Technologies, 9139). Goat anti-mouse and anti-rabbit secondary antibodies were used at 1:1000 dilutions (catalog numbers A11034, A11011, A11031, and A11001; Invitrogen, Thermo Fisher Scientific). For nuclear counterstaining, DAPI (1:1000; MilliporeSigma) was used. Coverslips were mounted using ProLong Gold Antifade Mountant (Thermo Fisher Scientific).

### Image acquisition.

Analysis of immunostaining on PDX brain tissue sections was performed on confocal stacks (with a step size of 1.5 μm) acquired with either a 10× or 20× water immersion objective on a laser-scanning confocal microscope (Leica Microsystems; TCS SP5) operated with LAS software. The fraction of nuclear STAT3 in vitro was quantified using the Intensity Ratio Nuclei Cytoplasm Tool on ImageJ (NIH; 5–8 images were processed for each experimental condition across 4 independent experiments). Fluorescence intensity quantifications of STAT3 in PDX tissue were performed on Fiji. Otsu’s method (https://imagej.nih.gov/ij/plugins/otsu-thresholding.html) was used to threshold engrafted GFP-positive glioma cells and create a selection. The mean gray values of STAT3 pixel intensity within the selected areas were then measured (4 to 5 images were quantified per mouse, with 3 mice total in each experimental group).

### Statistics.

Data are presented as the mean ± SEM. If comparing 2 conditions or cell lines, significance was tested with unpaired 2-tailed Student’s *t* test. Significance of the differences between conditions or cell lines were tested by the 2-way ANOVA with Bonferroni’s multiple-comparison tests using GraphPad Prism 8 (GraphPad Software). Survival studies were analyzed using the Kaplan-Meier method with the Mantel-Cox log-rank test (GraphPad Software). Statistical significance was defined at *P < 0.05, **P < 0.01, ***P < 0.001, ****P < 0.0001.

### Study approval.

GBM patients gave informed consent for the use of their tumor tissue for laboratory research. All patient samples used for this research were provided by the Biobank Core Facility at St. Joseph’s Hospital and Medical Center and BNI. The samples were deidentified and conformed to the Biobank Institutional Review Board–approved protocol. Animal husbandry was performed in accordance with the guidelines of the St. Joseph’s Hospital and Medical Center and BNI under the protocol approved by the Institutional Animal Care and Use Committee.

## Author contributions

CLC and SM conceived and designed all experiments. CLC standardized the techniques, performed in vitro and in vivo experiments, and analyzed the data. JBM performed in vitro experiments, assisted with in vivo experiments, and generated the lentiviruses. ELM performed in vivo experiments and immunohistochemistry experiments. EML and CLP performed all RNA-Seq and bioinformatic analyses. MED assisted with in vivo experiments. JBM, ELM, MED, EML, NS, and CLP edited the manuscript. NS provided patient tissues to establish patient-derived glioma cell lines. SM coordinated the project and helped analyze the data. CLC and SM wrote the manuscript.

## Supplementary Material

Supplemental data

## Version 1 09/08/2021

Electronic publication
