# Supplementary material for: Nonredundant, isoform-specific roles of HDAC1 in glioma stem cells
Source: JCI Insight. 2021 Sep 8;6(17):e149232. doi: 10.1172/jci.insight.149232 (PMC8492336; doi:10.1172/jci.insight.149232)
Supplement: Supplemental data [file jciinsight-6-149232-s054.pdf]

Cell Line	*TP53* Genotype
**BT70**	p.A273C
**BT187**	DelR283
**GB82**	c.822_825delTTGT
**GB84**	p.Cys277
**HK211**	p.N235S / p.Y234C
**HK252**	p.P250L

Supplemental Table 1. *TP53* mutations in the human GSC line used in this study (Related to Figure 1 and Supplemental Figure 2).

Cell Line	Genotype
**BT145**	*INK4a/ARF*$^{-/-}$ *; PTENmut/mut ; TP53*$^{+/+}$
**BT286**	*EGFRvIII; TP53*$^{+/+}$
**GB3**	*INK4a/ARF*$^{-/-}$ *; CDK6* gain *; CDK4* amp*; TP53*$^{+/+}$
**GB16**	*INK4a/ARF*$^{-/-}$ *; CDK6* gain *; CDK4* amp*; TP53*$^{+/+}$
**GB71**	*INK4a/ARF*$^{-/-}$ *; EGFR* gain *; CDK6* gain *; PTENmut/mut ; TP53*$^{+/+}$
**BT187**	*PTENmut/mut; P53 mut/mut (*DelR283)
**BT70**	*INK4a/ARF*$^{-/-}$ *; PTENmut/mut ; TP53mut/mut (*p.A273C)*; EGFR* amp *; EGFRvIII*
**GB82**	*INK4a/ARF*$^{-/-}$*; EGFR* amp *; CDK6* gain; *TP53mut/mut (*c.822_825delTTGT)
**GB84**	*INK4a/ARF*$^{-/-}$*; MDM4* loss*; RB1* loss*; PDGFR* loss*; EGFR* amp*; PTEN mut/mut ; CCND2* loss*; CDK4* loss*; MDM1/2* loss *; MDM4* loss *; FOXM1* loss; *; TP53 mut/ mut (*p.Cys277)
p16/p19$^{-/-}$***;EGFRvIII***	*Ink4a/ARF*$^{-/-}$ *; Olig2*$^{+/+}$ *; p53*$^{+/+}$ *;* h*EGFRvIII*

Supplemental Table 2. Human GSC lines used in this study (related to Figure 1 and Figure 4).

Cell Line	P-value	M	N	n	k	k genes
**BT145 up**	**0.002642433**	**27462**	**632**	**81**	**6**	**FAS, HMOX1, MAP3K8, CXCL10, A2M, CSF1**
BT145 down	0.492148388	27462	558	81	1	IL1B
**BT187 up**	**0.024730538**	**27462**	**973**	**81**	**6**	**BAK1, HMOX1, IRF1, PIM1, INHBE, PDGFC**
**BT187 down**	**0.012796318**	**27462**	**1059**	**81**	**7**	**CD9, CD44, LEPR, IL13RA1, TNFRSF21, CCR1, A2M**
**ihNPC up**	**9.05E-08**	**27462**	**939**	**81**	**14**	**TNFRSF12A, FAS, HMOX1, EBI3, TGFB1, IRF1, IL1B, IL6ST, IL15RA, PIM1, CXCL3, CXCL11, JUN, CSF1**
ihNPC down	4.91E-01	27462	557	81	1	TNFRSF21
NHA up	0.724877191	27462	859	81	1	HMOX1
**NHA down**	**0.000555088**	**27462**	**1152**	**81**	**10**	**CD38, IFNGR1, EBI3, IL18R1, LEPR, IRF1, TNFRSF21, CCR1, CD14, LTB**
**M = all genes in genome (from res files)**						
**N = differentially expressed genes**						
**n = genes from STAT3 pathway**						
**k = overlap between n & N**						

Supplemental Table 3. STAT3 Pathway Downstream Enrichment Analysis in BT145, BT187, ihNPCs and NHAs after HDAC1 knockdown (related to Figure 5). Downstream targets of the IL6/JAK/STAT3 pathway were examined for differential expression using a hypergeometric distribution model. Bolded cell lines (BT145 upregulated, BT187 upregulated, BT187 downregulated, ihNPC upregulated, NHA downregulated) indicate a significant difference in expression of the group of target genes, suggesting influence from the STAT3 signaling pathway.

Supplementary Methods

Live Bioluminescence (IVIS) Imaging

6 weeks post-implantation, the mice were examined for tumor growth by monitoring bioluminescence every 7 days using the IVIS Xenogen Spectrum platform. D-Luciferin Potassium Salt (Gold Biotechnology) was dissolved in PBS at a final concentration of 15 mg/mL. All mice were weighed each week and were administered D-Luciferin via an intraperitoneal injection (10µl/g). 15 minutes after the injection, the mice were sedated using gaseous isoflurane (Piramal) and placed inside an IVIS Spectrum In Vivo Imaging System (Perkin Elmer) for bioluminescence imaging. The total flux (photons/second) within the region of interest (ROI) was calculated using the Living Image Software 4.5 (Perkin Elmer).

Tissue Processing for Immunofluorescence

Moribund animals were sedated with an intraperitoneal injection of Ketamine (Henry Schein)-Xylazine (100 mg/kg, 10 mg/kg respectively). Intracardiac perfusions were performed with Ringer's solution (Electron Microscopy Sciences; 11763-10) supplemented with 40 mM NaNO2, 2 mM NaCHO3, and 50 IE/mL heparin, followed by ice-cold 4% paraformaldehyde (Sigma-Aldrich) in 0.1 M phosphate buffer (PB). The brains were manually extracted and placed in 4% paraformaldehyde for a further 24 hours at 4°C for post-fixation and dehydrated in 30% sucrose for 2 days. All brains were subsequently sectioned into 40 µm coronal sections using a vibratome (Leica VT1000 S; Leica Microsystems). Coronal sections were preserved long term at -20 °C in a 25% glycerol, 30% ethylene glycol, 45% 0.1M PB solution.

Chromatin Immunoprecipitation

hGSCs with 1% formaldehyde (Sigma) were fixed for 10 min at room temperature. Glycine (125mM) (Fisher Scientific) was added for 5 min at RT to quench formaldehyde. Cells were rinsed with PBS and Protease inhibitors (Pierce, 88266) twice. Cells were pelleted and flash frozen. Cells were sonicated in 50ul of SDS lysis buffer. For each immunoprecipitation, 30µl protein A Dynabeads (Invitrogen) were incubated with 2 µg of H3K27ac antibody (Abcam) or 1µg of IgG antibody for at least 4 hours at 4°C, and 15µg of sheared chromatin was pre-cleared with 15µl of protein A Dynabeads and incubated at 4°C for 1 hour. The pre-cleared chromatin was subsequently incubated with the antibody-coated beads at 4°C overnight. Beads were washed 6 times with RIPA wash buffer and twice in tris-EDTA. Reverse cross-linking was performed by incubating the beads with 100µl of reverse crosslinking buffer (1% SDS, 0.1M NaCl and 0.1M NaHCO3) overnight at 65°C. The immunoprecipitated DNA was purified using the QIAquick PCR purification kit (QIAGEN) and eluted in ddH2O. Chromatin-immunoprecipitated DNA was analyzed by quantitative PCR using the SYBR Green master mix in a real-time PCR system (Applied Biosciences). The following primers were used(57), for the CEBP/β binding site on the *STAT3* promoter: Stat3_1501_f: 5'-CAGGAGGGAGCTGTATCAGG-3' and Stat3_1630_r: 5'-AGGACTTGGGCACAGAAGC-3'.

Real-Time PCR

Total RNA was extracted from cells by using the PureLink RNA Mini Kit (Ambion) in accordance with the manufacturer's instructions. RNA was quantified on a NanoDrop Spectrophotometer (Tecan), and 1 µg of total RNA was used for cDNA synthesis by using the SuperScript VILO kit (Life Technologies). qPCR was performed using inventoried TaqMan assays for respective target genes and housekeeping control genes (18S) on the QuantStudio 6

Flex Real-Time PCR System (Life Technologies). Fold change in gene expression was analyzed using the delta delta Ct method.

Supplemental Figure 1.

A.

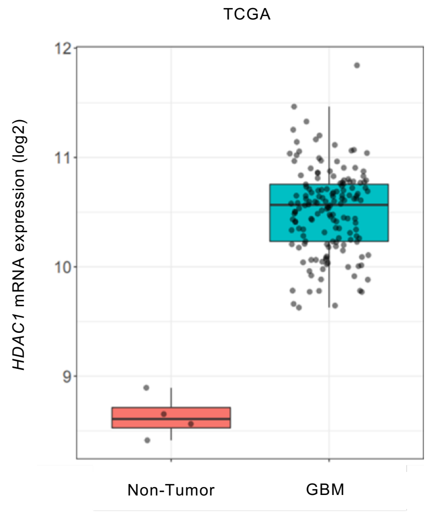

B.

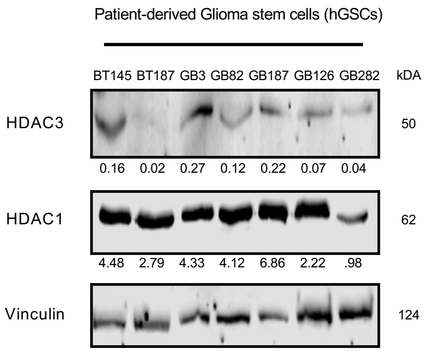

Supplemental Figure 1. Related to Figure 1. (A) Comparison of *HDAC1* expression levels from the TCGA dataset in GBM tissue and normal brain tissue from healthy controls. (B) Immunoblot comparing basal levels of HDAC1 and HDAC3 protein across seven different hGSCs lines. Numbers below the bands indicate normalized expression levels of HDAC3 and HDAC1 in each cell line relative to Vinculin (n=3). *** $p < 0.001$. Error bars indicate SEM.

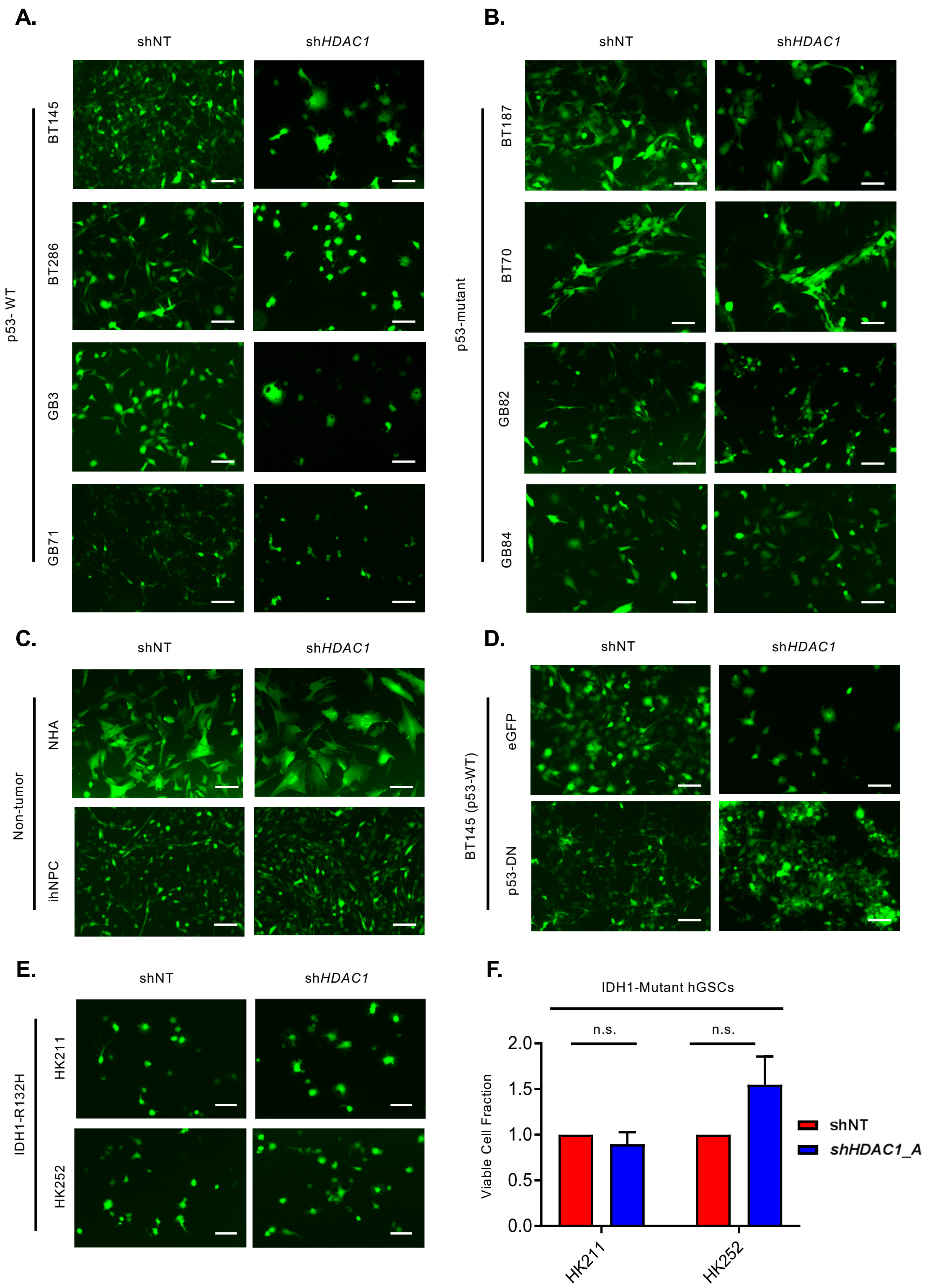

Supplemental Figure 2. Related to Figure 2. Knockdown of *HDAC1* impairs viability of human glioma stem cells (hGSCs) in a p53-dependent manner. (A-E) Representative images of control and *HDAC1*-silenced cells (GFP-positive) in (A) four p53-WT hGSCs, (B) four p53-mutant hGSCs, (C) non-tumorigenic NHAs and ihNPCs, (D) a p53-WT hGSC line (BT145) overexpressing a dominant-negative mutant of p53 (p53-DN) or a control vector (eGFP), and (E) two IDH1-mutant hGSCs. (F) Quantification of the viable fraction of *IDH1*-mutant hGSCs after HDAC1 knockdown compared to control cells transduced with shNT. For each cell line, the data are compiled from at least three independent experiments. Magnification 5x; scale bars, 2 μM; n.s., not significant. Error bars indicate SEM. *P* values were calculated using unpaired 2-tailed t-test.

Supplemental Figure 3.

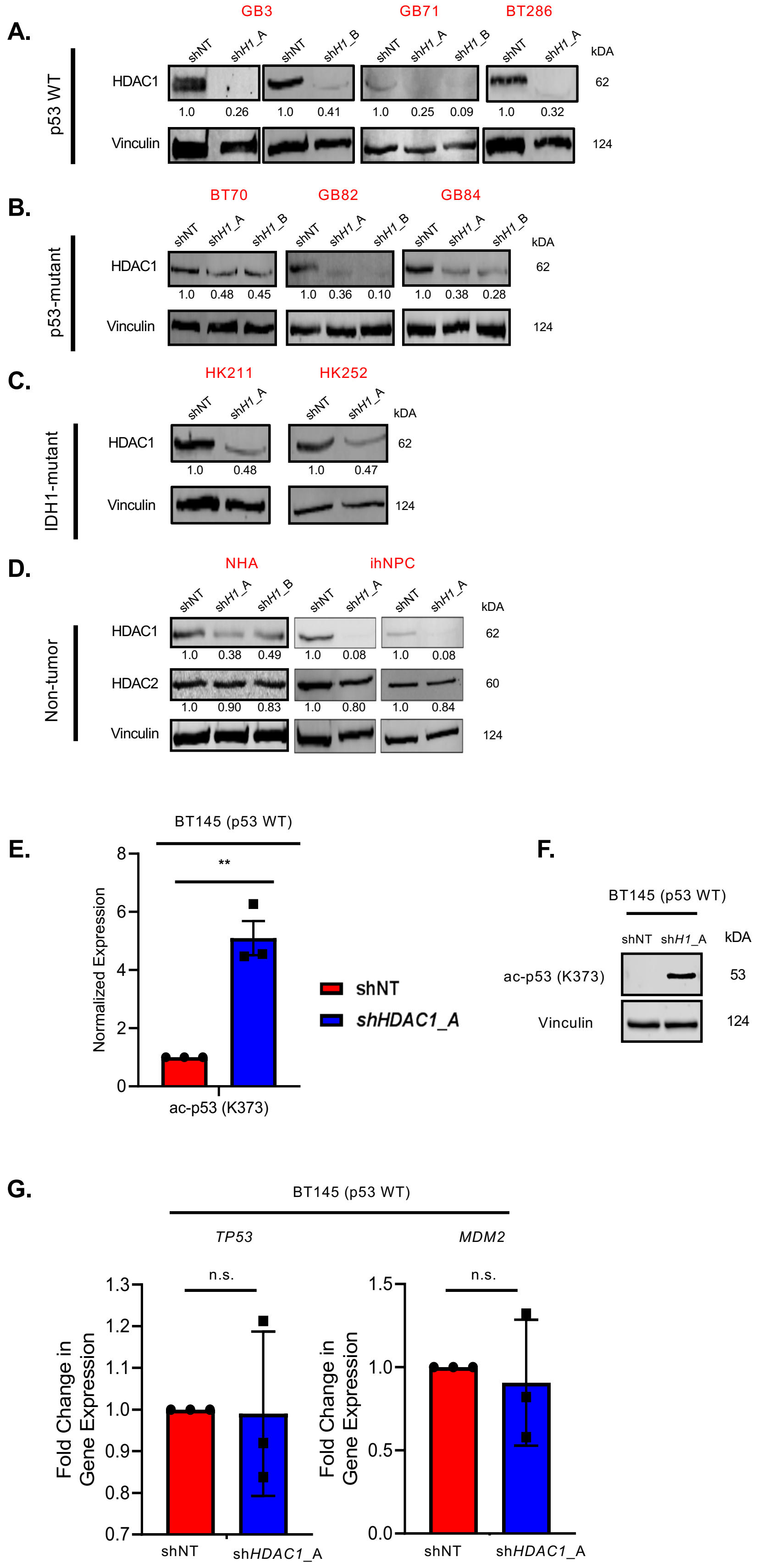

Supplemental Figure 3. Related to Figure 2. Knockdown Efficiency of *HDAC1* in hGSCs and relevant putative mechanisms of p53 activation. (A-D) Immunoblots showing *HDAC1* knockdown in (A) p53-WT hGSCs (B) p53-mutant hGSCs, (C) IDH1-mutant hGSCs and (D) non-tumorigenic NHAs and ihNPCs utilized for all cell viability assays shown in Figure 2. Numbers below the bands indicate normalized expression levels of HDAC1 and HDAC2 in each cell line relative to Vinculin. (E) Quantification of immunoblots for acetylated p53 (K373) after *HDAC1* silencing (sh*H1*_A = sh*HDAC1*_A) in p53-WT BT145 (n=3). (F) Representative immunoblot for p53 K373 acetylation after *HDAC1* silencing in p53-WT hGSCs (BT145). (G) RT-qPCR shows that *TP53* and *MDM2* are not differentially expressed in BT145 (p53 WT) hGSCs between control (shNT) and *HDAC1*-silenced cells (sh*HDAC1*_A, n=3). For each cell line, the data are compiled from three independent experiments for each shRNA. ** $p < 0.01$, n.s., not significant. Error bars indicate SEM. *P* values were calculated using unpaired 2-tailed t-test.

A.

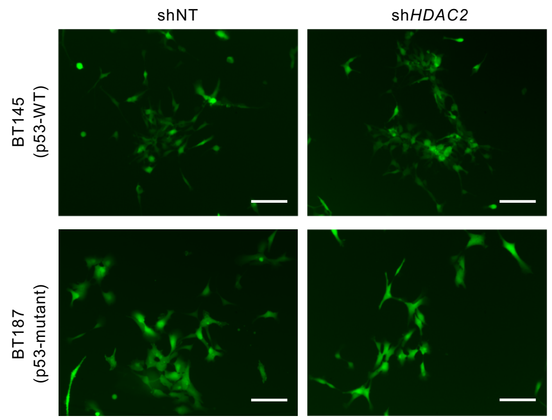

B.

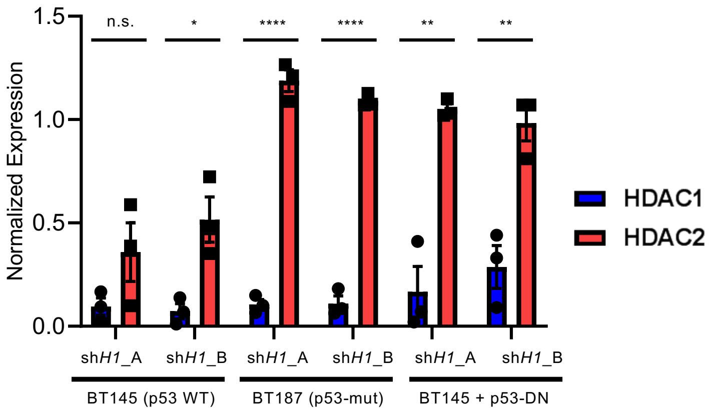

Supplemental Figure 4. Related to Figure 2 and Figure 3. Effects of HDAC2 knockdown and quantification of the normalized total levels of HDAC1 and HDAC2 proteins in hGSCs after *HDAC1* knockdown. (A) Representative images of control and *HDAC2*-silenced cells (GFP-positive) in p53-WT (top panel) and p53-mutant (bottom panel) hGSCs. (B) Quantification of expression of HDAC1 and HDAC2 protein levels after *HDAC1* knockdown in p53-WT, p53-mutant and p53-WT + p53-DN hGSCs (n=3). For each cell line, the data are compiled from at least three independent experiments for each shRNA. Error bars indicate SEM. * $p < 0.05$, ** $p < 0.01$, **** $p < 0.0001$, n.s., not significant. Magnification 5x; scale bars, 2 µM. *P* values were calculated using unpaired 2-tailed t-test.

Supplemental Figure 5.

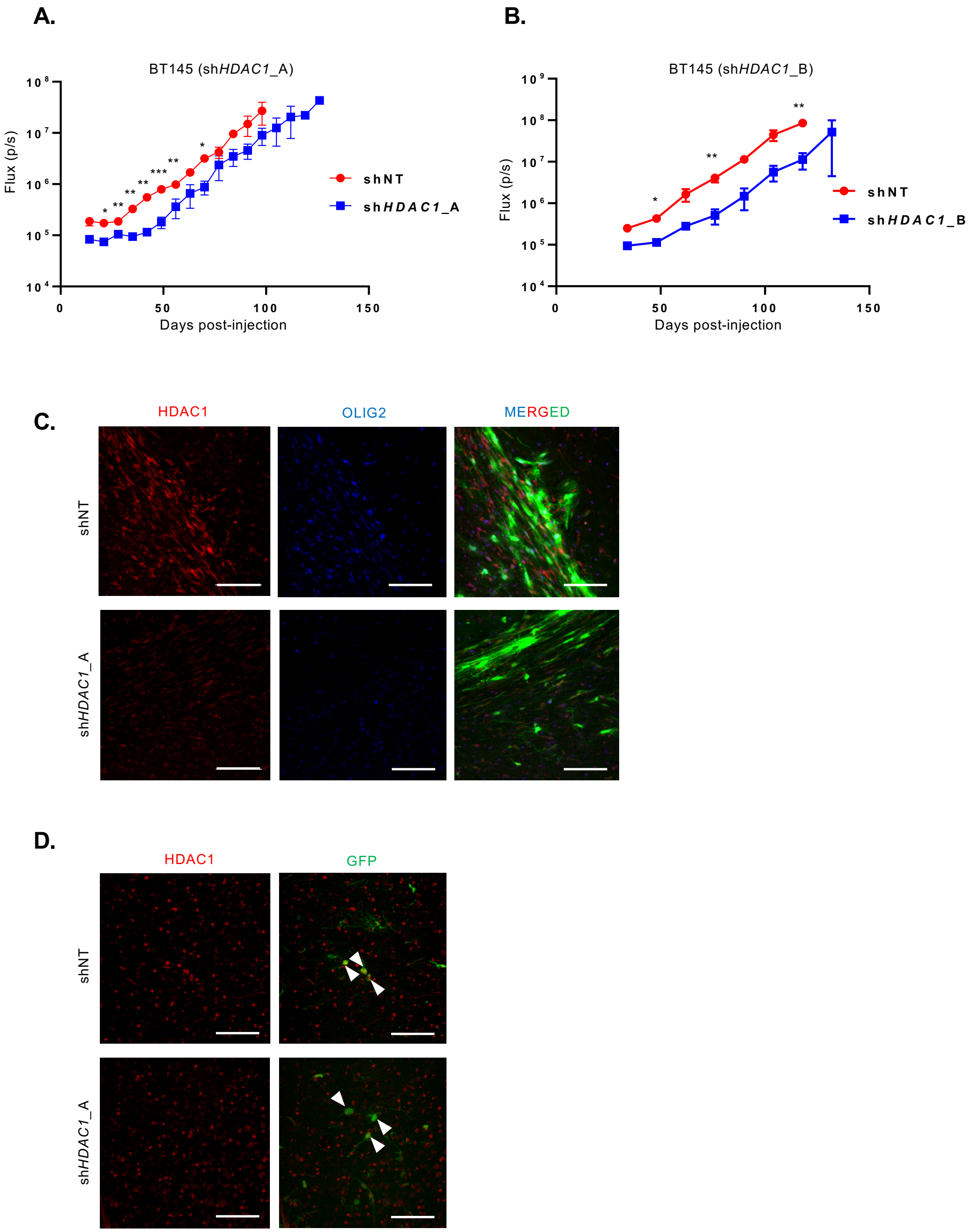

Supplemental Figure 5. Related to Figure 5. Bioluminescence imaging of shNT and sh*HDAC1* tumors. (A and B) Average photon flux (p/s) measured through bioluminescence imaging over time of mice with intracranial injections of shNT (n=4 in both experiments) and HDAC1-targeting shRNAs: sh*HDAC1*_A (n=4) and sh*HDAC1*_B (n=3). (C) Image of HDAC1 (red) and OLIG2 (blue) immunostaining in shNT and sh*HDAC1* BT145 tumor tissue 7 weeks post-injection (tumor core). (D) Image of HDAC1 (red) immunostaining in shNT and sh*HDAC1* BT145 tumor tissue (GFP) 7 weeks post-injection in the tumor leading edge (invasive front). Error bars indicate SEM. * $p < 0.05$, ** $p < 0.01$, *** $p < 0.001$. Magnification, 20x; scale bars, 100 μM. *P* values were calculated using unpaired 2-tailed t-test.

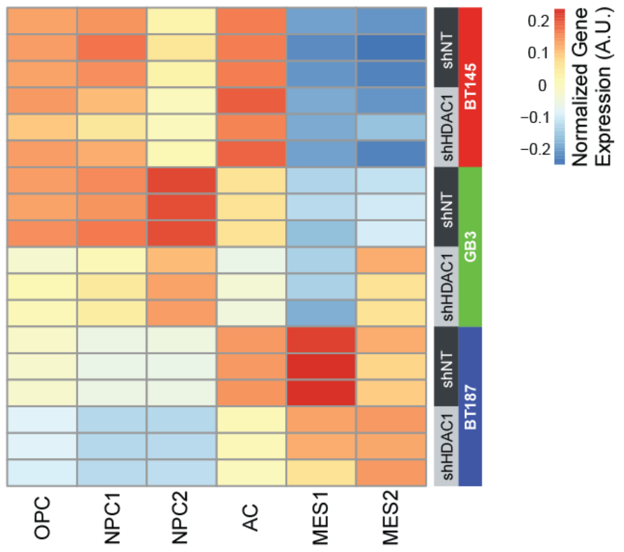

Supplemental Figure 6. Related to Figure 6. HDAC1 knockdown does not significantly affect the cellular states of hGSCs. Eigengene expression for the Neftel et al.,[25] developmental subtypes across the GSC cell lines after transduction with either shNT or sh*HDAC1*. Developmental subtypes include oligodendrocyte precursor cells (OPC), neural precursor cells (NPC1 & NPC2), astrocyte (AC), mesenchymal (MES1 & MES2).

Supplemental Figure 7.

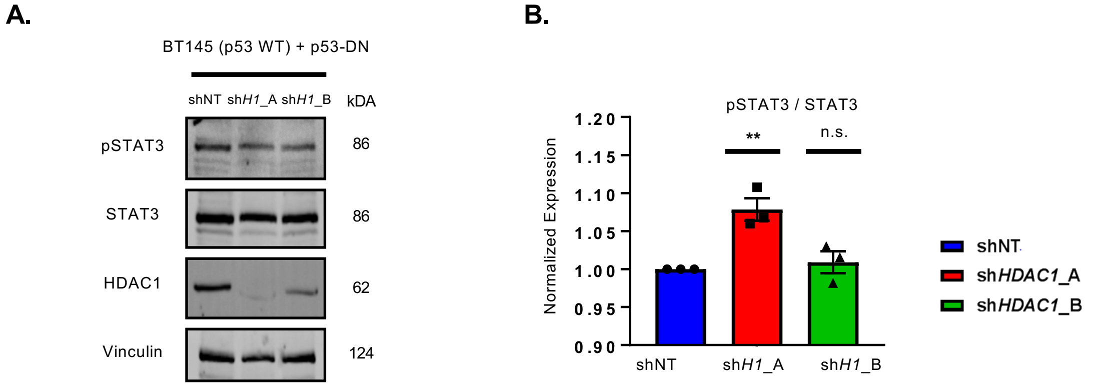

Supplemental Figure 7. Related to Figure 7. (A) Lysates were collected from BT145 overexpressing p53-DN after acute silencing of *HDAC1* with two independent shRNAs (sh*H1*_A = sh*HDAC1*_A and sh*H1*_B = sh*HDAC1*_B) and were immunoblotted with antibodies directed against phosphorylated STAT3 (Tyr705), STAT3, HDAC1 and Vinculin. (B) Quantification of the normalized ratio of pSTAT3 over total STAT3 protein after *HDAC1* knockdown from three independent experiments in BT145 overexpressing p53-DN. ** $p < 0.01$, n.s., not significant. Error bars indicate SEM. *P* values were determined using the 2-way ANOVA with Tukey's multiple comparisons test.
